# Public health partnerships with faith-based organizations to support vaccination uptake among minoritized communities: A scoping review

**DOI:** 10.1371/journal.pgph.0002765

**Published:** 2024-06-05

**Authors:** Melodie Yunju Song, Denessia Blake-Hepburn, Anna Karbasi, Shaza A. Fadel, Sara Allin, Anushka Ataullahjan, Erica Di Ruggiero

**Affiliations:** 1 Dalla Lana School of Public Health, University of Toronto, Toronto, Canada; 2 Clinical Public Health Division, Dalla Lana School of Public Health, University of Toronto, Toronto, Canada; 3 Institute of Health Policy, Management, and Evaluation, Dalla Lana School of Public Health, University of Toronto, Toronto, Ontario, Canada; 4 School of Health Studies, Faculty of Health Sciences, Western University, London, Canada; 5 Social and Behavioural Health Sciences Division, Dalla Lana School of Public Health, University of Toronto, Toronto, Canada; University of Michigan, UNITED STATES

## Abstract

Faith-based vaccine initiatives are of growing interest to public health agencies who are looking to increase vaccine confidence among ethnoracially minoritized populations. Despite evidence that support faith-based organizations’ (FBOs) partnerships with public health agencies (PHAs) to increase vaccine confidence, reviews on the scope and efforts to ensure equitable vaccination delivery for ethnoracially minoritized populations are scarce. We aimed to understand how public health agencies collaborate with FBOs or faith communities to improve vaccine confidence among minoritized communities in high-, low- and middle- income countries. We conducted a scoping review by searching OVID MEDLINE, Cochrane Library, Cumulative Index to Nursing and Allied Health Literature (CINAHL), SCOPUS, and PROQUEST from 2011 to 2023. We included case studies, news reports, observational studies, experimental, and quasi-experimental studies and multimedia content that describes PHA-FBO partnerships that created vaccine initiatives for marginalized and minoritized communities. The data was extracted, summarized, and results were described narratively. We included 167 initiatives reported in 160 publications; 83.8% of the included articles were published between 2019 to 2023. The interventions carried out by PHA-FBO partnerships attempted to increase vaccine uptake using any or all the following methods. First, the initiatives provided digital and in-person platforms for interfaith learning and established training programs to empower faith leaders to become vaccine ambassadors. Second, the initiatives designed and disseminated education and awareness materials that aimed to be sensitive to religious and gender norms. Third, PHA-FBO partnered to apply equity and faith-based frameworks and provided wrap-around support to enable equitable vaccine access. Majority of the initiatives reported that PHA-FBO partnerships improved vaccine confidence and uptake (71.3%). About 22.2% of the initiatives reported quantitative outcomes post-intervention. PHA-FBO initiatives over the past decade increased vaccine uptake and acceptance among diverse ethnoracially minoritized populations. Reporting of faith-based initiatives are subject to publication bias and can be strengthened by examining more evaluation studies and establishment of key outcome indicators to critically appraise intervention outcomes.

## Introduction

Despite the benefits of vaccination, vaccine hesitancy, “the delay in acceptance or refusal of vaccination despite availability of vaccination services”, was declared a pressing global threat by the World Health Organization in 2019 [1–3]. Ethnoracially minoritized communities and populations—ethnic and racial groups who experience multiple forms of discrimination [4] have faced low vaccine confidence [5–7] due to a legacy of systemic racism and medical maltreatments [8–10]. While terms such as ethno-racial, hard to reach, vulnerable and/or minority populations continue to be used in the literature, we intentionally use ethnoracially minoritized. Minoritization is a socially constructed concept as opposed to a naturally occurring phenomenon [11]. A group being minoritized does not imply a numerical minority, but rather that they are actively socially excluded. It explicitly acknowledges the power differentials that allow for those who are minoritized to also be systemically oppressed, marginalized and othered [11].

Public Health Agencies (PHAs), defined as “institutions or organizations with the authority responsible for, or advocate for public health matters as its official mission and mandate” [12], find strength in partnering with Faith-Based Organizations (FBOs), “entities whose organizational control, expression of religion, and program implementation are tied to values and beliefs belonging to specific religious identities” [13] that jointly apply principles of inclusivity, flexibility, and trust in community to promote vaccines [14]. The need to evaluate contemporary approaches to address hesitancy and increase vaccine confidence is needed, given the increased global rates of hesitancy [15].

Traditionally, reviews on PHA-FBO partnerships to increase vaccine confidence and uptake have focused on interventions in low-and middle-income countries (LMICs) [16–19]. Olivier and colleagues from the Joint Learning Initiative on Faith and Local Communities examined 43 examples of faith community participation in immunization programs in LMICs [17, 18]. Another review conducted by the Faith Engagement Team (FET) of the US Agency for International Development’s (USAID’s) MOMENTUM Country and Global Leadership program collected 110 studies on evidence of local faith actors (LFAs)’ influence on community vaccine hesitancy [16], and its most recent review described PHA-FBO partnership strategies that may improve COVID-19 vaccine confidence in four Sub-Saharan countries [19]. In addition to written reviews, conferences and panel sessions hosted by the World Health Organization (WHO), United Nations Children’s Fund (UNICEF), and Gavi, the Vaccine Alliance (Gavi)–offered collaborative opportunities for FBOs to share how PHA-FBO partnerships increase vaccine confidence and uptake in the context of low-and-middle income countries (LMICs) and open dialogues on issues of governance in equitable distribution [20–22].

In high-income countries (HICs), the documentation of PHA-FBO partnerships for vaccine interventions for minoritized communities largely consist of case studies [23, 24], recommendations [25], and PHA-disseminated toolkits [26–28]. An exemplar is the “Model Practices Framework” developed by Emory University’s Interfaith Health Program, whose facilitation of FBO collaboration with the Centers for Disease Control and Prevention (CDC) and local Departments of Health increased influenza vaccine uptake [26, 29, 30], and later that of COVID-19 vaccines [31].

Reviews of FBOs as public health collaborators of publicly sponsored community-based initiatives for vaccine promotion are scarce. To date, we found a report commissioned by The National Academy of Sciences that synthesized 23 qualitative articles highlighting community partnerships with FBOs which influenced community decisions to vaccinate [32]. A recent systematic review of 37 studies showed FBO’s ability to establish trust, mitigate barriers, disseminate and sustain efforts, and tailor public health campaigns, but did not examine interventions specifically for minoritized communities [33].

Despite applications of PHA-FBO vaccine interventions within the HIC and LMIC contexts, respectively, gaps remain with the description and analysis of PHA-FBO partnerships for engaging minoritized populations; the characteristics and outcomes of vaccine interventions; evidence of the theories/models/frameworks used to support the design, implementation, and evaluation of the intervention; and appraisal of implementation science theories/models/frameworks [34]. Given all the above, there is a need to characterize the various partnerships and approaches to strengthen the evidence for informing PHA-FBO engagement to promote vaccine confidence and uptake among minoritized communities.

The overarching question guiding our scoping review is: “How do public health agencies collaborate with faith-based organizations or faith communities to improve vaccine confidence among minoritized communities in HICs and LMICs?” Our objectives are to (1) describe the role of partners and collaborators involved in carrying out vaccine initiatives; (2) outline intervention strategies and implementation frameworks guiding interventions; (3) and synthesize outcomes and evaluations of PHA-FBO vaccine initiatives.

## Methods

### Protocol

Guided by the Joanna Briggs Institute (JBI) Scoping Review Method [35], a scoping review was conducted to map how PHAs, FBOs, and other organizations collaborate to improve vaccine confidence among ethnoracially minoritized communities. Scoping reviews are useful for identifying evidence gaps and determining conceptual patterns that emerge in an emerging field of study [36]; it is a suitable knowledge generation methodology to capture faith-based collaboratives for vaccine. A comprehensive description of our methods is described in our published protocol [37]. We applied the PCC (population, concept, and context) approach to align study selection with our research question (See Table 1) [38]. The protocol is registered with Open Science Foundation (OSF Registration number: https://doi.org/10.17605/OSF.IO/DAX5Z).

**Table 1:** Population, concept, and context of search concepts.

	Search Concept
Population	Minoritized communities with low vaccine uptake due to reasons of vaccine hesitancy, knowledge deficit, barriers to access, and cultural and personal beliefs. Minoritized communities broadly encompass ethno-racial and vulnerable populations who experience health inequities due to historic and systemic racism and social and cultural oppression.
Concept	The concept of PHA-FBO partnerships for vaccine confidence and uptake refers to partnerships formed to design, implement, and evaluate interventions delivered to the population of concern, which involve PHAs, departments of health, ministries of health, FBOs, and religious and spiritual leaders. The interventions may be based on theories, models, and frameworks of implementation science for the promotion of vaccine confidence and uptake.
Context	The context of the intervention can occur in any geographic region in LMICs and HICs. The context can be virtual or in-person at a physical location.

### Inclusion and exclusion criteria

We included documents that capture interventions to increase vaccine confidence and uptake that are jointly carried out by PHAs and FBOs, using the PCC-anchored keywords and concepts related to intervention AND vaccine* AND *faith-based organizations* AND *public health agencies* (See S1 File).

#### Information sources and literature search

Comprehensive literature search was conducted from October 20, 2023 to October 30, 2023 on OVID MEDLINE, Cochrane Library, Cumulative Index to Nursing and Allied Health Literature (CINAHL), SCOPUS, PROQUEST-Public Health, PROQUEST-Conference Paper, PROQUEST- Policy File Index. Considering the changing landscape and wealth of community engagement approaches for vaccine uptake, only those published between January 1, 2011, and October 20, 2023, were searched. We included major vaccine preventable diseases eradication campaigns such as influenza (e.g., H1N1), polio, measles, and COVID-19.

The search strategy was piloted by two researchers (MYS and DB-H) and Medical Subject Heading (MeSH) terms were validated by a librarian using the PRESS checklist available in our protocol publication [37]. Hand searches in 11 document repositories for public health, 4 institutional archives, 8 websites, 6 journals, and 3 dissertation portals with substantive relevance in the implementation and research in faith-based initiatives and/or vaccination efforts were also performed. Finally, a Google Search Engine was performed to extract grey literature (e.g., reports, statements, media releases, webinars, and toolkits) for PHA-FBO partnerships from reputable news media, not-for-profit (.org), government (.gov), and research institutions (.edu,.ca). Reference scanning was performed on systematic reviews, review of reviews, and scoping reviews to determine if they contain relevant references that meet the inclusion criteria for extraction.

Detailed search strategies are documented in S2 File. All search records (academic and grey literature sources) are documented in S2 File and imported into Covidence for database management and extraction [39].

### Study selection process

Two researchers (MYS and DB-H) calibrated the inclusion/exclusion criteria using a random sample of 20 titles and abstracts screened independently by each researcher. Using Covidence, each researcher’s independent review was blinded to the other researcher. Weekly meetings were held to discuss and resolve differences in agreement and to clarify eligibility amongst the pair and a third member of the team (MYS, DB-H, TS). The title only progressed to full-text screening when the reviewers reached consensus.

Full-text screening was performed by four researchers (DB-H, MYS, TS, AK). Each document was screened by two researchers to progress to the extraction phase. We follow the checklist of the Preferred Reporting Items for Systematic Reviews and Meta-Analysis Extension for Scoping Review (PRISMA-ScR) [40].

### Data extraction and synthesis

A document extraction codebook was developed to extract relevant and consistent information from each document (See S3 File); each article was extracted by two researchers and validated by a third researcher.

To synthesize the included studies, we summarized the extracted information, including: (1) Document characteristics (i.e., type of publication, country economy, project name, year of publication); (2) Partners and their roles in the faith-based vaccine initiative (i.e., PHA partners, FBO partners, Other partners, and how these agencies collaborate with each other in the conceptualization, design, implementation, evaluation, and funding of the initiatives); (3) Main objectives and strategies of faith-based initiatives; including extraction of the study designs, models/theories/frameworks applied in the initiatives, religions and languages considered in initiative delivery, target population, recruitment process, initiative duration, number of participants recruited, number of vaccines administered, and participant characteristics; and (4) outcomes, to the extent available, including initiative results, findings, limitations, and future directions.

## Results

### Characteristics of included documents

**Fig 1:**
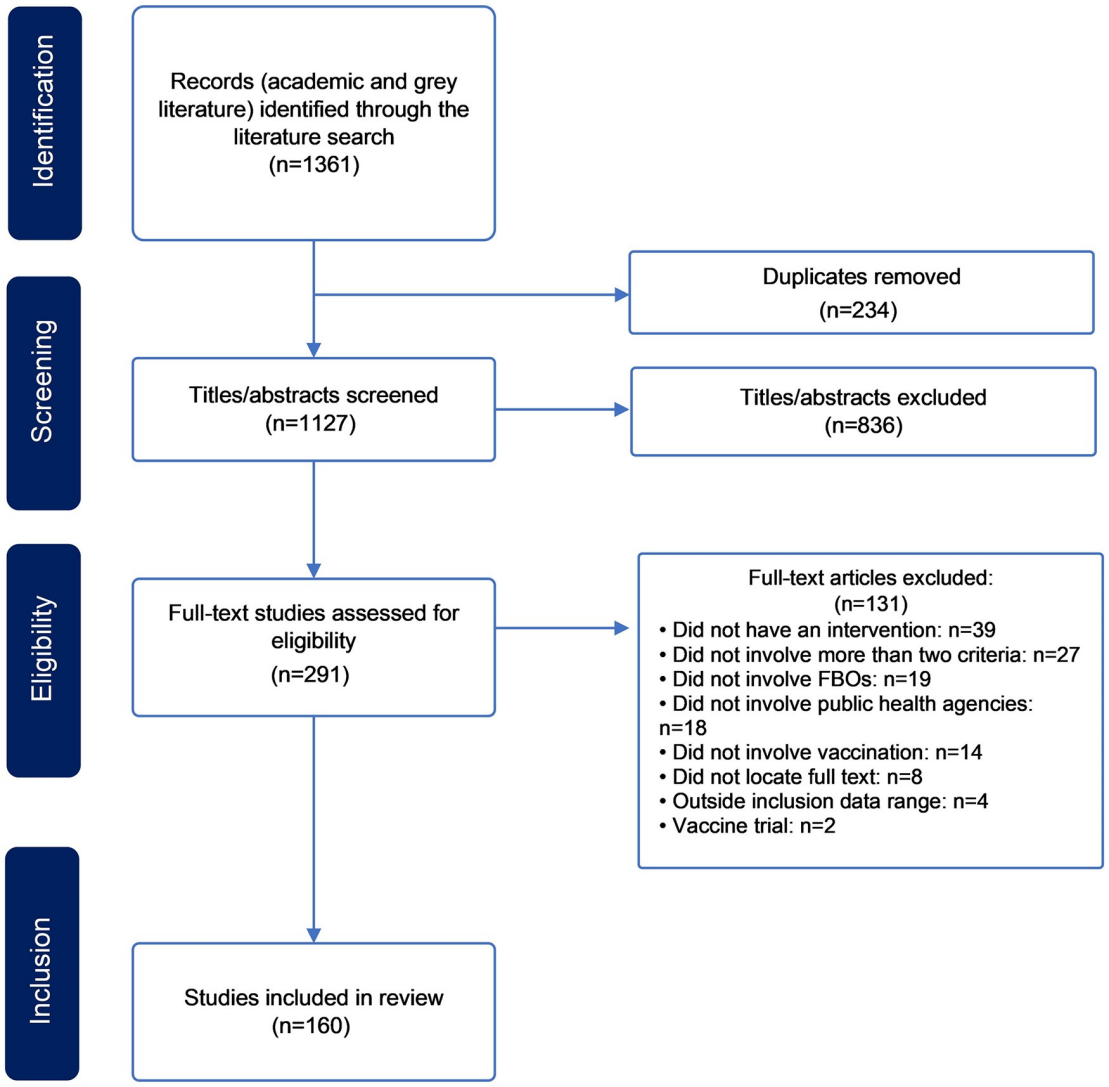
PRISMA ScR flowchart.

Our search yielded 1361 results; 160 articles were included in this scoping review for analysis (See Fig 1).

Most were published between January 2019-October 2023 (134/160, 83.8%), while 26 articles (16.3%) were published between January 2011-December 2018; 167 initiatives were described based on 160 included articles. The number of initiatives is not the same as the number of documents collected, because one article may describe multiple initiatives (See Table 2).

**Table 2:** Grey literature characteristics.

		Count`	(%)
**Year of publication**	2011–2014	15	9.4
	2015–2018	11	6.9
	2019–2023	134	83.8
	**Total**	160	100
**Publication type**	Journal: PH	42	26.3
	Media article	39	24.4
	Journal: Medicine	16	10.0
	Journal: Social Science	5	3.1
	Journal: Multidisciplinary	5	3.1
	Other	53	33.1
	**Total**	160	100
**Country economy**	High Income Countries (HICs)	101	63.1
	Low- and Middle-Income Countries (LMICs)	30	18.8
	Multiple Countries	16	10
	Global	13	8.1
	**Total**	160	100
**Funding source**	Government^1^	49	30.2
	Not Reported	45	27.8
	NGO/INGO	23	14.2
	University/Research^2^	23	14.2
	Co-sponsorship or multiple sponsorship^3^	17	10.5
	Faith Based Organizations	4	2.5
	Industry	1	0.6
	**Total** ^4^	162	100

^1^ Including publicly funded public health agencies and hospitals.

^2^ Includes government research grants, institutional funding, and donation of funds directly *for* non-partisan research-oriented institutions for the knowledge production and dissemination.

^3^Any sponsorship involving more than 2 types of organizations. Example: Government and NGO, NGO and faith-based organizations, government and research sponsored, together.

^4^The total number of funding sources is more than the total amount of articles included (n = 160) because some articles include multiple initiatives with different funding sources.

COVID-19 vaccinations were the most common vaccines promoted among initiatives (122/199), followed by childhood vaccines (17/199), and polio vaccines (15/199). The target populations were mainly faith communities (78/332), ethno-racial and minority populations (50/332), and vaccine hesitant populations (36/332) (See Table 3).

**Table 3:** Target populations and vaccines promoted.

Intervention Population	Count
Faith-based populations	78
Ethno-racial and minority populations	50
Vaccine hesitant populations	36
Public health professionals, healthcare provider, and frontline workers	33
Underserved and hard-to-reach populations (e.g., unhoused, transient, inaccessible, et cetera)	30
Rural populations	25
Immigrants	22
Low-income populations	19
People living in high-conflict zones	8
People living with disability	8
Underprivileged urban populations	8
Refugees	6
Women	4
**Total**	**327** ^1^
**Intervention by Age-Groups**	
Children (0–11)	26
Adolescents (12–18)	11
Adults (18–65)	10
Seniors (65+)	18
Not specified	118
**Total**	**183** ^2^
**Vaccines administered**	
COVID-19 vaccines	122
Childhood vaccines^3^	17
Polio vaccines	15
Measles, Mumps, and Rubella (MMR) vaccines	12
Influenza (HiB) vaccines	12
Human papillomavirus (HPV) vaccines	4
Hepatitis B vaccine	3
Diphtheria, tetanus, pertussis (DTap) vaccines	3
Rotavirus (Rotavax) vaccines	2
Pneumococcal vaccines	2
Other vaccines^4^	3
Not Specified	4
**Total**	**199** ^5^

^1^Initiatives (n = 167) may target more than one target population.

^2^Initiatives (n = 167) may target more than one age-group.

^3^Include initiatives promoting multiple childhood vaccines that did not the specify vaccine type.

^4^Other: Avian flu (n = 1), Meningitis (n = 1), Pentavalent vaccines (DPT, HepB, HiB) (n = 1).

^5^Each initiative (n = 167) may promote/administer more than one kind of vaccine.

**Fig 2:**
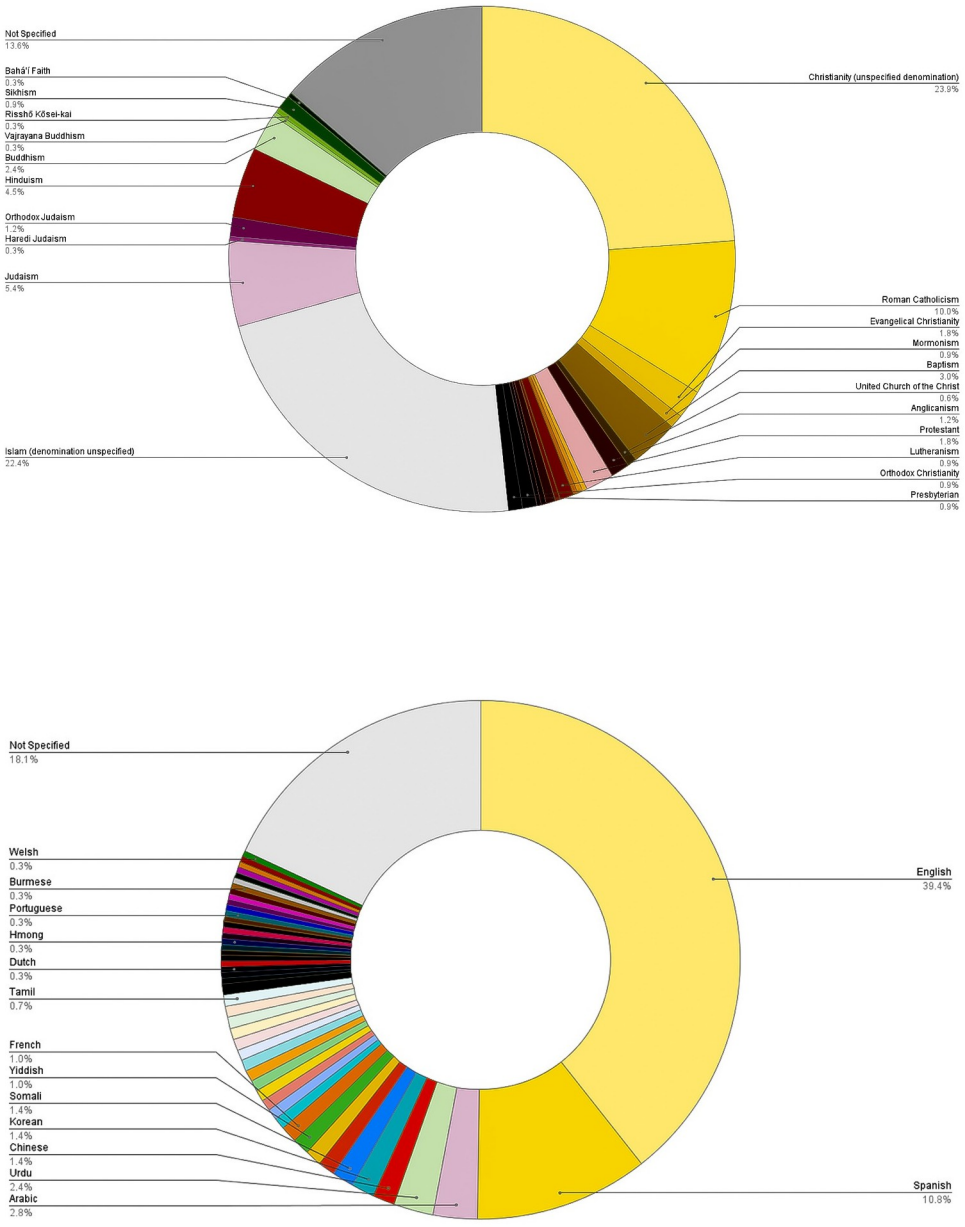
Religions involved in vaccine initiatives (2A); Languages applied in vaccine initiatives (2B).

The initiatives were carried out among 7 identified religions, including Christianity (160/331, 48.3%) and Islam (74/331, 22.4%) (See Fig 2A). Initiatives were delivered in 48 languages, majority in English (113/288, 39.2%), Spanish (31/288, 10.8%), and Arabic (8/288, 2.8%), as well as other languages such as Bedouin, Temne, Hmong, and Tongan (See Fig 2B) (See S4 File).

### Leadership among public health agencies (PHAs), faith-based organizations (FBOs) and other partners

PHAs, FBOs and other partners contributed to the implementation and delivery of initiatives to varying degrees. Initiatives were mainly co-led (37.7%, 63/167) by PHAs, FBOs, and other partners. PHAs conceptualized and designed 34.7% (58/167) of the initiatives. Research institutions and universities led 12.6% (21/167) of the initiatives (See S5 File). FBOs led 8.4% (14/167) of the initiatives and community-based organizations (CBOs) led 3.6% (6/167), while 3.0% (5/167) of the initiatives did not specify lead partner(s). In terms of program delivery, FBOs accounted for 28.5% (97/340) of the delivery personnel, including religious leaders and representatives from a faith community. Public health/global health professionals comprised 16.5% (56/340) of the delivery personnel, while healthcare professionals (e.g., physicians, nurses, pharmacists) comprised 14.1% (48/340) of personnel involved in the delivery of initiatives (See S6 File).

There were 38 frameworks, theories, models and strategies applied across 44 initiatives (See Table 4). Six theories/frameworks/models/strategies were identified in more than 2 articles, namely, the Social-Ecological Model (SEM) [41–44]; Community Based Participatory Research (CBPR) [44–48]; Community-Engaged Outreach (CEO) [49, 50]; Social and Behavioral Change Communication framework (SBCC) [41, 51]; Social Mobilization Implementation Theory and Strategy [52, 53]; and Consolidated Framework for Implementation Research (CFIR) [43, 54].

**Table 4:** Frameworks/model/theories/strategies used, and corresponding target population and vaccines promoted.

Theories, frameworks, models and strategies applied	Intervention demographic	Vaccines	N	References
The Social Ecological Model (SEM)	Faith communities in LMICs; Marshallese communities in Arkansas, USA	Childhood vaccines; COVID-19 vaccines	4	[41–44]
Community Based Participatory Research (CBPR)	Christian Korean Americans in Texas, USA; Children in rural communities in India; Christian Marshallese and Hispanic communities in rural Arkansas, USA, Underserved Black community in New York, USA	HepB vaccines; COVID-19 vaccines, HiB, Seasonal Influenza	4	[44–48]
Community-Engaged Outreach (CEO)	Vulnerable communities—older adults, those with preexisting conditions, racial and ethnic minorities, those with disabilities, and other vulnerable populations, Nevada, USA; Black communities, USA	COVID-19 vaccines	2	[49, 50]
Social and Behavior Change Communication (SBCC)	Pregnant women and mothers with children in Ethiopia; African American church community in Georgia, USA	Childhood vaccines, HPV vaccines	2	[41, 51]
Social Mobilization Implementation Theory and Strategy	Areas with low coverage rate and low knowledge of vaccines in LMICs	Childhood vaccines (e.g., MMR)	2	[52, 53]
Consolidated Framework for Implementation Research (CFIR)	The Haitian-Creole community, the Cape Verdean community, the Latino community, the Black Christian Faith community, guardians who care for children living with disabilities, and individuals affected by systemic lupus erythematosus in the Boston area, Massachusetts, USA; Polio endemic LMICs, conflict-affected areas, communities of faith	COVID-19 vaccines, Polio	2	[43, 54]
Model Practices Framework	Vulnerable, at-risk communities, minority populations, USA	HiB	1	[14, 26, 30]
Behavioral and Social Drivers of Vaccination Framework	Global faith communities, trusted FBO/faith leaders	Not specified	1	[55]
Coalition Advocacy Framework	Global leaders of faith-inspired organizations	Childhood vaccines^1^	1	[22]
Community Engagement Framework (Ahmed and Palermo, 2011)	African American/Black, American Indian/Alaska Native, and Hispanic/Latinx community members Christian and Catholic communities in Arizona, USA	COVID-19 vaccines	1	[56]
Community Health Worker (CHW) model	People from diverse faith backgrounds reaching out to their religious and ethnic communities	COVID-19 vaccines	1	[57]
Conceptual framework of the drivers of vaccine delivery (Philips et al, 2017 & LaFond et al, 2015)	Minority, religious groups, rural communities in Zambia	DTap vaccines	1	[58]
Cross-cultural Collaborative Model	Faith-based African American communities in California, USA	COVID-19 vaccines	1	[59]
Framework for community engagement in interventions (Brunton et al., 2017)	Black and Latino residents, seniors, younger adults, essential workers, Haitian community, Cape Verdean community, local businesses and groups in highly vulnerable urban areas in Massachusetts, USA	COVID-19 vaccines	1	[60]
Health Belief Model	Black parents who are hesitant or unaware of HPV-induced cancers and vaccines in south central North Carolina, USA	HPV vaccines	1	[61]
Health-Interfaith Community-Based Organization (CBO) COVID-19 Prevention Model; the Risk Communication Community Engagement (RCCE) Framework	Religious leaders, hard-to-reach vulnerable groups, general population in Sri Lanka	COVID-19 vaccines	1	[62]
Human-Centered Design (HCD)	Caregivers of under-immunized children in underserved and hard-to-reach locations in Nigeria	Childhood vaccines	1	[63]
Locally Driven Partnerships and Community-designed Solutions	Low-income groups, hard-to-reach groups in the USA	COVID-19 vaccines	1	[64]
Operational Framework of Community Engagement through Faith-Based Organizations	Faith leaders across all provinces of India	COVID-19 vaccines	1	[65]
Social Identity Theory	Vaccine hesitant Christians in USA	COVID-19 vaccines	1	[66]
Stakeholder Analysis	Local religious leaders that are influential among vulnerable populations to reach hard-to-reach and vaccine hesitant groups in Uttar Pradesh, India	Polio	1	[67]
Taskforce on Racial Inclusion & Equity (TRIE) framework	Marginalized and underserved communities, Black and Latinx communities in New York, USA	COVID-19 vaccines	1	[68]
The 3R (Reframe, Reprioritize, and Reform) Communication Model	Parents/caregivers and their 12–17-year-old adolescents from three local churches in the Ashanti Region of Ghana	HPV vaccine	1	[69]
The 5C psychological antecedents of vaccination (“5C”) model	Parents, community health workers, religious figures and leaders in Zambia, Nepal, and Senegal	Childhood vaccines	1	[70]
The Core Group Polio Project (CGPP) Secretariat Model	Transient mobile/seasonal workers of Muslim faith in LMICs	Polio	1	[71]
Vaccine Hesitancy Determinants and Matrix of Vaccine Hesitancy	Vaccine hesitant African American faith community in Missouri, USA	COVID-19 vaccines	1	[72]
The Global Polio Eradication Initiative (GPEI) “Endgame" Strategy—community engagement	Areas with low polio vaccine confidence in North Nigeria	Polio	1	[73]
The National Response Framework (NRF) for Emergency Management	Areas with high social deprivation in the USA	COVID-19 vaccines	1	[74]
Training-of-Trainers (ToT) framework	Community members, correctional officers, and incarcerated jail residents in Westchester, New York	COVID-19 vaccines	1	[75]
Trauma Informed Community Development	Church pastors to serve underserved, marginalized, at-risk communities, Black residents in Pennsylvania USA	COVID-19 vaccines	1	[76]
The Supporting the Use of Research Evidence (SURE) Framework	Faith communities with low polio immunization rates in Nigeria	Polio	1	[77]
The Theological Moral Framework of the Good Samaritan	Hispanic Evangelicals with history of institutional low trust in science in the USA	COVID-19 vaccines	1	[78]
Theory of Change	Muslim majority state in north-western Nigeria with below-average immunization coverage	Childhood vaccines and polio	1	[79]
Community collaboration model; The Monitoring, Evaluation and Learning (MEL) Framework	Communities in underserved and rural locations, persons living with disability in India	COVID-19 vaccines	1	[80]
WHO Health Systems Framework; ‘Hit and Run’ Strategy	Polio endemic LMICs, conflict-affected areas, communities of faith	Polio	1	[43]

^1^ Childhood vaccination: Include more than one childhood vaccination for the following diseases (e.g., Hepatitis A, Hepatitis B, Rotavirus, Influenza, Haemophilus Influenza type B, diphtheria, tetanus, Streptococcus pneumonia, varicella, measles, mumps, rubella, acellular pertussis).

*Abbreviations: HIV: Human Immunodeficiency Virus; HepB: Hepatitis B Virus; HiB: Haemophilus Influenzae Type B Vaccine; HPV: Human Papillomavirus.

### Objectives and strategies of faith-based vaccine initiatives

Categorizing the initiatives by their intended objectives, faith-based vaccine initiatives mainly attempt to achieve the following: (1) enrolling faith leaders to support and promote vaccines, (2) increasing vaccine confidence among ethno-racial minorities, (3) increasing equitable access to vaccines. Strategies to achieve the objectives are described under each objective.

#### Objective 1: Enrolling faith leaders

Enrolling local faith leaders to support vaccines is one of the critical objectives that INGOs, faith-based humanitarian organizations, and social justice-oriented associations rely on to influence marginalized and vulnerable populations [42, 71, 81, 82]. Three main strategies to recruit faith leaders include facilitating interfaith learning; establishing vaccine training programs for faith leaders; and mobilizing faith leaders as vaccine ambassadors.

*Interfaith learning*. Interfaith learning was reported to accelerate knowledge exchange among diverse faiths via conferences, webinars, and in-person dialogues. Internationally, faith-based humanitarian societies (e.g., World Council of Churches (WCC) [83, 84], World Vision [83–85], Muslim Aid [22], Parliament of the World’s Religions [86], Network for Religious and Traditional Peacekeepers (NRTP) [87, 88], Religions for Peace (RFP) [86, 89, 90] worked with public-health oriented INGOs (WHO, UNICEF, Gavi). Multi-faith leaders congregated to discuss practical means to vaccinate communities in low-resource high-risk settings [22, 73, 86], communities facing language barriers [62], those experiencing gender and race oppression [88], statelessness [83], and residents in conflict zones (e.g., Israel/Palestine, Sri-Lanka, Sierra Leone) [62, 91, 92].

An example of global interfaith learning was the “*Communities of Practice (COPs)”* series hosted by WHO Information Network for Epidemics (EPI-WIN) which fostered continuous collaboration and dialogue among faith leaders with local knowledge on ways to approach vaccine hesitant communities in 5 different continents [93]. Second, WCC partnered with global public health and human rights agencies (i.e., United Nations High Commissioner for Refugees, WHO-EPI WIN, UNICEF, and Gavi) to reach vulnerable communities of various Christian denominations in 28 countries [83].

Regional PHA-FBO initiatives facilitated dialogues in conflict-ridden communities. For instance, the Sri-Lankan Health Promotional Bureau created an interfaith network with local FBOs and NGOs to promote vaccination and overcome prevailing stigmatization that Muslims spread COVID-19 [62]. In the US, nationwide high-level inter-ecumenical coalitions such as the “Faiths4Vaccines” formed by The National Council of Churches and the Network for Religious and Traditional Peacemakers ensured equitable COVID-19 vaccine distribution and access for racialized populations [87].

*Establishing training programs*. PHA-FBO initiatives focused on establishing vaccine training programs to empower faith leaders to become vaccine advocates and community ambassadors. Led by UNICEF [18, 53, 81], Cambodian monks and nuns were trained by UNICEF to join influenza campaigns [18]; similar programs were delivered to faith leaders in Angola, Ethiopia, India, Liberia, and Pakistan to deliver polio vaccine education in sermons and at in-home visits [53, 81], as well as engage in COVID-19 prevention efforts [94]. In HICs, the CDC and Association of State and Territorial Health Officials (ASTHO)—an organization representing the public health agencies of all 50 States and Territories in the US—participated in the “Engaging Communities in Response to Pandemic Influenza” project to engage FBOs in flu vaccine promotion, [95]; a survey revealed that ASTHO members that worked with FBOs viewed these partnerships as valuable for reaching ethno-racial minority groups [31].

FBOs with establishing networks such as World Vision and the Christian Relief demonstrated great capacity to lead PHA-FBO initiatives that recruit faith leaders for vaccine uptake [16, 96]. For instance, World Vision’s “Channels of Hope COVID-19 vaccine model” trained 8,000 Christian faith leaders of diverse denominations to design vaccine outreach strategies, resulting in a rapid mobilization of 400,000 faith leaders in 50 countries that reached rural and poor communities without television, radio, and internet access [19]. Regional FBOs like the Henry Ford Macomb Faith Community Nursing Network (FCNN), have offered training to parish nurses and lay health ministers, on how to address misinformation and connect people to social resources to improve vaccine confidence [97].

*Recruiting faith leaders as vaccine ambassadors*. Another strategy was to recruit leaders as vaccine ambassadors. The intensity of engagement ranged from consultation with faith-based leaders to establishing task forces, working groups, and collaborative frameworks that empower community decision-making. PHAs were often the leaders of this strategy. PHAs offered funding opportunities for FBOs to conduct vaccine outreach and education [98, 99]. PH -and collaborative network-led vaccine ambassador programs were observed in both HICs and LMICs [65, 95, 100–102], which included a training component to equip ambassadors with the knowledge to promote vaccination; a similar FBO-led program was observed within the LMIC context [58]. Student mobilizers of faith communities were capitalized to promote vaccination in both LMICs and HICs [67, 74, 81, 103, 104]. For instance, the “Faith in the Vaccine” ambassador program sponsored by The Interfaith Youth Core (IFYC) trained religious youths to engage with their own religious/ethnic communities [57, 105]. PHAs also funded faith leaders to build community relationships among immigrant communities [48, 106], and further bolster their work with vulnerable populations [107]. As seen in tight-knit faith communities, ethno-racial minorities, and marginalized communities [96, 108–110], vaccine confidence among African Americans [72], Korean immigrant church goers [46], minoritized Jewish populations in the United Kingdom [111], and Rohingya refugees in Pakistan [112, 113] increased.

#### Objective 2: Increasing vaccine awareness and acceptance among minoritized groups

PHA-FBO vaccine partnerships aimed to increase vaccine awareness and acceptance among communities that demonstrate mistrust towards the medical establishment, those who are uncertain whether their faith allows for vaccination, and ethno-racial minority groups with language/access barriers to vaccines and vaccine-related information. This objective builds on the first objective (enrolling faith leaders), because faith leaders’ receptivity and proactivity to collaborate with PHAs is a contributor of vaccine acceptance. Two strategies were used: awareness and education campaigns; and designing faith-sensitive vaccine communication.

*Awareness and education campaigns*. First, awareness campaigns were carried out via digital pathways. To generate educational materials, PHAs solicited the help of healthcare practitioners of faith (e.g., the American Muslim Health Professionals) [114] and dialogued with faith-based groups [115] to design and guide intervention efforts. Religious scholars endorsed and promoted vaccine safety and efficacy information from PHAs to mitigate hesitancy in their communities [45, 116–118], which was shown to improve communication of vaccine-related information and inspire willingness to receive the vaccine [45]. On more collaborative fronts, PHAs partnered with FBOs to launch multipronged educational campaigns via webpages, statements, webinars to combat myths and address safety and efficacy concerns from faith communities who mistrust government institutions and healthcare systems [116, 117, 119–121]. Faith-based digital media campaigns [122] and community-academic-faith-based partnerships were also shown to improve vaccination rates among targeted communities through the implementation of educational tools (i.e., coloured graphics that answer COVID frequently asked questions (FAQs)) [104].

Second, in-person gatherings in places of worship to promote vaccine confidence was used to approach communities with low vaccination literacy and vaccination rates [123]. Faith leaders partnered with local PHAs (e.g., Departments of Health, Ministry of Health) to promote vaccines in sermons [77, 81, 91, 97, 124, 125], which produced positive results [122]. “Pulpit swaps” allowed vaccine informed pastors to preach in other churches [19, 91]. Town hall events [115, 126–128], information sessions [129], Q&As [130, 131], and group meetings were promotional strategies used to encourage vaccination [54, 70, 81, 91], which was particularly useful for promoting trust among hesitant communities.

Third, toolkits designed by PHAs and government/research institutions took the form of implementation guidelines, outreach strategies, and vaccine promotion materials in multiple languages for FBOs to promote confidence among hesitant, vulnerable, at-risk, and ethno-racial minority populations [26, 27, 55, 132–136]. Some of these toolkits had community engagement models co-developed with local faith communities [91, 132, 137]. For instance, in Zanzibar, public health engaged religious leaders in a co-creation workshop to develop a toolkit that addresses COVID-19 concerns which became a key vaccination strategy [138].

Fourth, FBOS played significant roles in fostering safe spaces for Black and Latino communities within the U.S. Both communities have experienced marginalization and discrimination within the health system but have long-standing collaborations with local PHAs and continuous health ministering programs that PHAs have supported. Black-church networks like “Choose Healthy Life” and “Black Coalition Against COVID-19” strengthened its partnership with PHAs, colleges and universities to put out vaccination guidelines [139, 140]. An academic-community-public health partnership led to the development of a linguistically and culturally-sensitive educational book on COVID-19 for community health workers to distribute among Black/Latino communities [104]. In Latino faith communities, faith leaders developed vaccination education material to equip church-going elders and parents with credible information to advise younger loved ones who were less likely to attend church to get vaccinated [110].

#### Designing faith-sensitive vaccine communication

Faith leaders released statements jointly with PHAs to promote COVID-19 vaccines’ morally acceptability and encourage vaccinations [80, 139, 141–145]. Faith leaders vaccinated publicly in front of congregations [19, 107, 139, 146, 147] and answered concerns with an understandable perspective that incorporated faith [103, 115].

First, engaged religious leaders cited passages from religious or spiritual/sacred text to promote the protective benefits of vaccines [19, 43, 67, 90, 91, 113, 139, 148, 149]. Editors of widely circulated religious magazines were enrolled by Global Polio Eradication Initiative (GPEI) to produce polio-vaccine promotional content in alignment with readers’ faith [113]. Social media was utilized by religious leaders (e.g., imams, ultra-Orthodox rabbinical scholars and pastors) to respond to vaccine concerns with justifications abiding to religious scripture [65, 119, 120, 139].

Second, campaigns adhering to religious gender norms were implemented. In HICs, vaccine stations for Muslim women were established for newcomers [116]. In LMICs, gender-sensitive campaigns were used in 7 majority Muslim countries where leaders separately addressed men and women on parental obligations to vaccinate children based on sacred text [81]; and women ambassadors were trained to address women of the same faith to increase vaccination rates among children in communities where male family members are vaccine resistant [91].

Third, messaging was also tailored based on linguistic and cultural understanding [47, 109, 150]. The National Latino Evangelical Coalition (NLEC) worked with the CDC and Health and Human Services to promote Spanish public service announcements on digital platforms [78, 139]. Israeli civil society organization, Kavod engaged with faith-based health practitioners to approach vaccine resistant communities in the Hasidic Jewish and Arab communities in Yiddish and Hebrew, and in Arabic among Israeli Arab communities, leading to increased vaccination rates [92, 151].

Fourth, some FBOs led the design of vaccine communication protocols and operational frameworks that adhered to religious practice for PHAs [82]. For instance, the Islamic Civic Society of America (ICSA) provided a guideline for PHAs to engage FBOs on the fence about vaccines [82]. FBOs also led workshops for PHAs on their communities’ beliefs and vaccine concerns that improved vaccine acceptance [152].

Finally, in countries with a state religion, rapid vaccine coverage was observed when PHAs partnered with faith leaders affiliated to the state. In Bhutan, the King, Prime Minister, and the Health Minister heeded advice from Buddhist monks to delay first vaccination in accordance with the Buddhist calendar, resulting in the country immunizing 93% of its adult population in 2 weeks and reaching 66% of the total population [153]. In polio endemic Afghanistan, the GPEI attributed increasing vaccination rates to the effort of the Department of Religious Affairs who distributed polio vaccine promotional briefs to mosques [43].

#### Objective 3: Increase equitable access to vaccines

The success of faith-based NGOs is rooted in addressing vaccine accessibility from equity-based approaches. At the G20 Global Health Summit, the Global Solidarity Fund (GSF) and COVID-19 Vaccine Global Access (COVAX) partners coordinated a *“Vaccines for All”* event, leveraging its network of 700,000 Catholic Sisters in 26 countries to identify and redirect vaccine delivery to regions where scale-ups and delivery were difficult and inequitable [154].

First, places of worship served as focal points for PHAs to establish a transparent and accessible platform to disseminate vaccine reminders for passive/uninformed members of the community. In LMICs, faith communities’ vast volunteer networks were activated to facilitate vaccine appointment sign-ups [111, 155, 156]; and mosques and churches were staffed to call its members to show up at vaccine appointments [81, 91, 113] while also being used as vaccination sites [80]. In HICs, vaccine clinics were established at locations and times convenient to vulnerable ethno-racial communities, which were useful for addressing language barriers [44, 47, 64, 102]. Coalitions of PHAs, FBOs, and civil society NGOs organized mass vaccination sites at places of worship and vaccine drives. Longstanding PHA-initiated programs included the Minnesota Immunization Networking Initiative (MINI) [157] and White House Faith-Based and Neighborhood Partnerships [87]. Newer programs like the multi-faith "Faith 4 vaccines" initiative [158], National Black Church Initiative (NBCI) [109], National Latino Evangelical Coalition (NLEC) [78], Nevada Vaccine Equity Collaborative [159], and university-PH-FBO collaborative in Arkansas, US [44, 47] were exemplars in developing an equitable vaccination distribution plan and ensuring vaccine sites met the linguistic and cultural needs of the community. Data-driven strategies were used to determine areas with high social deprivation index disproportionately impacted by COVID-19 [74, 87].

Second, FBOs have long played an important role in hard-to-reach, war-torn and conflict zones [96, 160]. The Core Group Polio Project (CGPP) enrolled imams, pandits, and priests as partners of the Social Mobilization Strategy (SMS) to deliver polio vaccination. In war-torn Afghanistan, all health services were outsourced, such that local FBOs worked under political constraints and were funded by the government to immunize locals (ibid). In Pakistan, the Basic Integrated Rural Development Society’s immunization program worked on remote areas of Khyber Pakhtunkhwa [91]. In Ethiopia, the CGPP enlisted key informants to connect with nomadic pastoralists whose mobile population’s children are at high risk of polio to get vaccinated [81]; a similar method was also employed in Nigeria under the same global program [43].

Third, wrap-around services improved vaccine access among racial minorities, vulnerable, low-income, immigrant communities, and other groups like at-risk pregnant and young mothers, people experiencing homelessness, and transient populations in HICs. Coalitions such as the Interfaith Health Program [30]; the Muslim Association of Canada [116], the Edmonton COVID-19 Rapid Response Collaborative [161], and the Magen David Adom (MDA) [151], provided transportation, social services, food, shelter, and other income support for vaccine seekers; similar efforts were observed in LMIC context [145].

#### Reporting of outcomes

Majority of initiatives reported that faith leaders improved vaccine uptake and confidence (119/167, 71.3%); these were mainly reported through subjective interviews, media reports, case studies, organizational reports, and webinars.

Three initiatives reported that the interventions produced mixed outcomes [76, 123, 151]. For instance, the state religion Romanian Orthodox Church had pockets of parishes with influential leaders who vocally opposed COVID-19 vaccines despite performing various charitable work for pandemic relief [123]. Three initiatives did not report successful intervention outcomes and expressed obstacles in dispelling vaccine misinformation among certain religious organizations such as the Muslim Rights Concern (MURIC) and the Nigerian Christ Embassy Church in Nigeria [88] and in two branches of Orthodox Protestant religious minority groups in the Netherlands [162]. One initiative reported low uptake in a Nigerian state due to mistrust of the COVID-19 vaccines [94].

A total of 37 initiatives reported positive quantitative intervention outcomes (22.2%). These included 12 quasi-experimental community trials [53, 62, 63, 69, 71, 73, 91, 96, 113, 153, 163, 164], 12 surveys [31, 57, 88, 105, 119, 122, 131, 137, 158, 165–167], 5 randomized controlled trials [46, 66, 79, 124, 168], 5 case studies [19, 110, 125, 169, 170], 2 mixed methods studies [43, 52], and one program evaluation [171]. A total of 42 initiatives reported number of participants reached and 26 reported number of vaccines administered (See Table 5).

**Table 5:** Outcomes reporting of the initiatives.

Reported Outcomes	N (%)	Initiatives
Reported pre-post outcomes (cohort study, cross-sectional study, cross-case comparisons, quasi-experimental, cross-sectional surveys, randomized control trials, economic analysis, etc)	37/167 (22.2%)	[19, 31, 43, 46, 52, 53, 57, 62, 63, 66, 69, 71, 73, 79, 88, 91, 105, 110, 113, 119, 122, 124, 131, 153, 158, 163–166, 168, 170–175]
Reported number of participants reached	42/167 (25.1%)	[30, 48, 57, 60, 61, 63, 64, 75, 78, 80, 91, 98, 100–102, 104, 109, 111, 112, 122, 127, 129, 143, 149–151, 155, 157, 166, 169, 170, 176–183]
Reported number of vaccinations administered	26/167 (15.6%)	[31, 59, 60, 63–65, 68, 74, 80, 92, 94, 100, 102, 104, 122, 138, 139, 146, 147, 150, 156, 159, 169, 170, 184, 185]
Reported subjective outcomes from interviews	50/167 (29.9%)	[14, 22, 31, 41, 42, 44, 45, 50, 52, 54, 62, 65, 67, 71, 72, 76–78, 81–83, 88, 94, 97, 100, 101, 116, 121, 126, 128, 138, 142, 144, 146, 150, 152, 154, 157, 162, 167, 186–194]
Reported no outcomes	42/167 (25.1%)	[47, 55, 56, 58, 61, 64, 70, 82, 86, 90, 94, 98, 99, 103, 106–109, 114, 118–120, 130, 132, 133, 136, 140, 143, 145, 161, 167, 175, 178, 191, 195–203]

## Discussion

Our study shows that PHA-FBO partnerships have increased, with over half occurring after 2015 and particularly during COVID-19. To note, the concepts guiding our search are guided by past reviews on the role of FBOs collaborating with public health partners to advance immunization initiatives in LMICs [17, 18]. We further expanded the literature search to incorporate initiatives in HICs that were aimed at increasing vaccine confidence and uptake among ethnoracially minoritized communities. The reviewed studies suggest that setting broader parameters (i.e., search terms) for “minoritized communities” yielded substantial literature involving initiatives that are aimed at communities and populations from diverse racial and ethnic backgrounds.

Our review reveals that most initiatives were started and funded by governmental PHAs and public health international non-governmental organizations (INGOs). We also found that FBOs take on proactive leadership positions to identify areas in need and to organize vaccine communication and outreach that adhere to cultural and religious norms. The reviewed studies confirm that PHAs benefited from FBOs’ localized understanding of the needs and concerns of ethnoracially minoritized communities [204]. First, PHAs (e.g., WHO, UNICEF, USAID) partner with FBOs (e.g., Religions For Peace, World Faiths Development Dialogue, Catholic Relief Services, World Vision, World Jewish Congress) to deliver interfaith learning to empower faith leaders to network and become ambassadors to their own communities [45, 91, 146, 189]; faith leaders were recruited to advisory councils which lead to stronger commitments to participate in vaccine promotion digitally [79, 122, 142] and in-person [97, 171]. Second, PHA-FBO partners co-created vaccine awareness and educational materials and campaigns like “the Color Green Vaccination” [188], “Build Hope Together” [88] with careful consideration to religious beliefs and local practices, thereby contributing to minoritized communities’ acceptance and understanding of vaccines. Finally, improving equitable vaccine access is one of the many priorities of FBOs that PHAs have leveraged to create longstanding impacts to address the needs of communities made structurally vulnerable such as ethnoracially minoritized groups [205, 206]. Leveraging FBOs’ shared commitment in providing humanitarian aid [55], wrap-around services [162], and social supports [111] to marginalized communities in both LMICs [16] and HICs [207] was seen as instrumental to the success of these partnerships.

Among initiatives that reported frameworks, models, and strategies, PHAs did not explicitly consider the socio-structural drivers of racial and health inequities. Rather, they took on community-centered implementation approaches (e.g., CBPR [44–48]; CEO [49, 50], Social Mobilization Implementation Theory [52, 53], Community Engagement Framework [56], Locally-Driven Partnerships and Community-designed Solutions [64] etc.). Local faith-based partners’ understanding of cultural and social norms and contextual needs, and as established and trusted leaders within their respective communities positively informed vaccine outreach activities. A few applied vaccine hesitancy models/frameworks (e.g., The 5C psychological antecedents of vaccination [70], Behavioral and Social Drivers of Vaccination Framework [55], Vaccine hesitancy Determinants and Matrix of Vaccine Hesitancy [72]). Very few explicitly centered equity, applied intersectional approaches when analyzing relationships between FBOs and PHAs (e.g., CFIR [43, 54], Health Interfaith CBO COVID-19 prevention model [62], etc.) or problematized the socio-structural influences of minoritization impacting different ethno-racial groups (i.e. Taskforce on Racial Inclusion and Equity framework [68]).

Our study has answered the call for evidence-informed approaches, in collaboration with faith-based communities that narrow vaccine delivery equity gaps among ethnoracially minoritized communities [23]. The review addresses a knowledge gap on the use of faith-based partnerships to improve equitable vaccine distribution among various low vaccine uptake communities, including zero-dose children [208, 209], migrant populations [183], newcomers and refugees [210], and rural communities [211]. FBOs provided PHAs the tools (i.e., faith-based frameworks) to engage and network with communities in rural, remote, or conflict-ridden regions of the world where CBOs and PHAs previously had difficulty accessing vaccines, demonstrating FBOs’ potential impact on equitable vaccine delivery [187, 212].

Despite these contributions, our scoping review highlights limitations in the ways that FBOs’ contributions are not well-documented. The findings of this scoping review, echoing others [213], point to a critical need to further evaluate the impact of initiatives.

### Limitations

We note a few limitations for the scoping review. First, the study only included interventions reported in English. Second, in keeping with scoping review methodology, we did not assess the effectiveness of strategies for reaching ethnoracially minoritized population groups who are historically made vulnerable by oppressive structures (e.g., different strategies that worked for Indigenous Peoples, refugees in conflict zones). Third, this paper may be subject to some publication bias given the inclusion of media reports and organizational reports that primarily showcase the positive impacts of PHA-FBO partnerships on vaccine confidence and uptake. Further research is needed into the utility of standardized performance indicators to avoid positively skewed outcomes among PHA-FBO partnerships whose reporting may be influenced by the need to secure continued funding for sponsors.

## Conclusion

PHA-FBO vaccine initiatives improved vaccine uptake among faith-based communities and ethnoracially minoritized populations who are made structurally vulnerable due to racism, statelessness, stigmatization, oppression and/or marginalization. Collaborations between PHA-FBO and other partners promoted vaccine confidence and access by interfaith learning and training, awareness and education with faith-based sensitivities, and wrap-around services to increase accessibility based on principles of equity. Future research on the impacts of faith-based initiatives can be strengthened using theories/conceptual frameworks that help to interrogate and analyze the historical and sociopolitical processes of minoritization, complemented by implementation science methods to assess the processes and outcomes of implementing interventions and their health equity impacts.

## Supporting information

S1 File Inclusion and exclusion criteria. (DOCX)

S2 File Scoping review search strategy and outcome. (DOCX)

S3 File Document extraction codebook. (DOCX)

S4 File Religions and languages of vaccine initiatives. (DOCX)

S5 File Public health partners, faith-based organization partners, and other partners. (DOCX)

S6 File Lead partners and vaccine delivery personnel. (DOCX)
