# Supplementary material for: Public health partnerships with faith-based organizations to support vaccination uptake among minoritized communities: A scoping review
Source: PLOS Glob Public Health. 2024 Jun 5;4(6):e0002765. doi: 10.1371/journal.pgph.0002765 (PMC11152308; doi:10.1371/journal.pgph.0002765)
Supplement: S1 File — (DOCX) [file pgph.0002765.s001.docx]

# Supporting Information 1. Inclusion and Exclusion criteria

**Inclusion criteria:**

1. Must involve ministry of health, public health agencies, department of health, health agencies, community health centres, or any government-funded health unit/department/agency.
2. Must involve religious entities, faith leaders, faith-based organizations, spiritual leaders of any faith, as well as government sponsored institutes and organizations that provide service to all faith.
3. Must involve interventions related to improving vaccination uptake, including any infectious diseases awareness program that have a vaccine promotion component (e.g., HIV education programs to increase vaccine trial willingness).
4. Must be published in English literature.

**Exclusion criteria:**

1. Not published in English.
2. Literature published between January 1 2011 and October 20 2023.
3. Commentaries, opinion pieces, meta-analysis, systematic reviews, review of reviews.
