# Supplementary material for: Public health partnerships with faith-based organizations to support vaccination uptake among minoritized communities: A scoping review
Source: PLOS Glob Public Health. 2024 Jun 5;4(6):e0002765. doi: 10.1371/journal.pgph.0002765 (PMC11152308; doi:10.1371/journal.pgph.0002765)
Supplement: S2 File — (DOCX) [file pgph.0002765.s002.docx]

# Supporting Information 2: Scoping review search strategy and outcome

**Databases**: OVID MEDLINE, Cochrane Library, CINAHL, SCOPUS, PROQUEST-Public Health, PROQUEST-Coronavirus Research Database, PROQUEST - Policy File Index, Thesis Canada, PROQUEST – Dissertations & Thesis, Networked Digital Library of Theses and Dissertations, Center for Infectious Diseases Research and Policy, UMN , Repository of Public Health Evidence Synthesis, Health Canada, National Collaborating Centres for Public Health (NCCPH), Canadian Best Practices Portal, Public Health Agency of Canada (PHAC), Canadian Institute for Health Information (CIHI), Rural Health Information Hub, Joanna Briggs Institute COVID-19 Special Collection, Can COVID Resources & Events, Custom Search Engine for Ontario Public Health Unit Websites (OPHLA), Google Scholar (a search engine)

**Search terms**:

1. vaccination: (free text terms: vaccin*, vaccine hesitan*, vaccine confidence, vaccine acceptance, vaccine refusal, vaccine uptake, anti-vacci*, anti-vaxx*, antivaxx*, antivacc*, immuniz*, immunis*)
2. Faith-based organization: (faith-based agenc*, faith-based communi*, faith-based organization*, faith-based leader*, spiritual*)
   - Religion: Christian*, Catholic, Protestant*, Evangelical*, Lutheran, Islam*, Muslim, Sunni, Shia, Orthodox, Judaism, Sikh*
   - Places of worship: church*, mosque*, temple*, synagogue*
   - Religious clergy: priest*, nun*, monk*, deacon*, bishop*, mullah, rabbi
3. Public health agencies: (community health centre*, public health units, public health agencies, ministry of health, indigenous health centre*, aboriginal health access centre*)

**Inclusion**: English, published between Jan 2011- Oct 2023

**Exclusion**: commentaries, opinion pieces, meta-analysis, systematic reviews, review of review

## **Detailed search strategies**

### **Theses repositories**

**1 Thesis Canada**

| **Search query** | **Date searched** | **# results retrieved** | **#results selected** |
| --- | --- | --- | --- |
| "(vaccin* OR immuniz*) AND (religio*) AND ("confidence" OR "promotion" OR uptake)" | 2023-10-16 | 29 | 0 |

**2 PROQUEST – Dissertations & Thesis** (<https://about.proquest.com/en/dissertations/>)

| **Search query** | **Date searched** | **# results retrieved** | **#results selected** |
| --- | --- | --- | --- |
| "(vaccin* OR immuniz*) AND (religio*) AND ("confidence" OR "promotion" OR uptake)" | 2022-03-07 | 7 | 7 |
| (abstract(vaccin*) OR abstract(immuniz*)) AND (abstract(faith*) OR abstract(religio*)) | 2023-10-16 | 17 | X |

**3 Networked Digital Library of Theses and Dissertations** (<http://search.ndltd.org/>)

| **Search query** | **Date searched** | **# results retrieved** | **#results selected** |
| --- | --- | --- | --- |
| description: "vaccine confidence" OR "vaccine hesitancy" OR "immunization" AND Orthodox OR church OR synagogue OR Temple AND "community engagement" OR "public health" | 2023-10-16 | 75 | 16 |

### **Grey literature repositories for Public Health**

### **1 Center for Infectious Diseases Research and Policy, UMN,** (<https://www.cidrap.umn.edu/>)

| **Search query** | **Date searched** | **# results retrieved** | **#results selected** |
| --- | --- | --- | --- |
| Faith | 2023-10-16 | 10 | 8 |
| Faith OR religious | 2023-10-16 | 10 | 3 |

**2 Repository of Public Health Evidence Synthesis** (<https://www.nccmt.ca/covid-19/covid-19-evidence-reviews>)

| **Search query** | **Date searched** | **# results retrieved** | **#results selected** |
| --- | --- | --- | --- |
| vaccin*, faith  (<https://www.nccmt.ca/covid-19/covid-19-evidence-reviews?q=vaccin*%2C+faith>) | 2023-10-16 | 52 | 13 |

**3 Health Evidence (**<https://www.healthevidence.org/>**)**

| **Search query** | **Date searched** | **# results retrieved** | **#results selected** |
| --- | --- | --- | --- |
| ((vaccin* OR immuniz*) AND (intervention OR effectiveness OR implementation) AND (faith* OR religio* OR muslim OR shrine* OR temple* OR synagogue OR mosque* OR church* OR christian OR buddhis* OR orthodox* OR indigenous*)) | 2023-10-16 | 6 | X |

**4 National Collaborating Centres for Public Health (NCCPH)** (<https://nccph.ca/resources/)> & **National Collaborating Centre for Indigenous Health** (<https://www.nccih.ca/en/>)

| **Search query** | **Date searched** | **# results retrieved** | **#results selected** |
| --- | --- | --- | --- |
| vaccin* OR immuniz* AND (faith OR relig* OR church OR mosque OR christian OR belief OR temple OR hindu* OR sikh* OR buddhis*) | 2022-03-08 | 7 | 1 |
| Spirit* OR faith* OR belief* AND (Vaccin* OR immuniz*) | 2023-10-16 | 24 | 7 |

**5 Canadian Best Practices Portal** (https://cbpp-pcpe.phac-aspc.gc.ca)

| **Search query** | **Date searched** | **# results retrieved** | **#results selected** |
| --- | --- | --- | --- |
| (Vaccination OR immunization) AND (religious OR faith) | 2022-03-22; 2023-10-16 | 0 | 0 |

**6 Health Canada, Public Health Agency of Canada, and other Federal Government** (<https://www.canada.ca/en/sr/srb.html?_charset_=UTF-8&idx=0&q=faith%2C%20vaccination%2C%20immunization#wb-land>)

| **Search query** | **Date searched** | **# results retrieved** | **#results selected** |
| --- | --- | --- | --- |
| vaccin* OR immuniz* AND faith | 2023-10-16 | 318 | 7 |

**7 Canadian Institute for Health Information (CIHI)** (<https://www.cihi.ca/en/search?query=faith&Search+Submit=>)

| **Search query** | **Date searched** | **# results retrieved** | **#results selected** |
| --- | --- | --- | --- |
| Immunization OR vaccination OR vaccine | 2023-10-16 | 79 | 1 |
| Faith | 2023-10-16 | 6 | 0 |

**8 Rural Health Information Hub (**<https://www.ruralhealthinfo.org/resources/topics/community-and-faith-based-initiatives>)

| **Search query** | **Date searched** | **# results retrieved** | **# results selected** |
| --- | --- | --- | --- |
| Vaccin*, faith | 2023-10-16 | 41 | 13+ |

**8 Joanna Briggs Institute COVID-19 Special Collection** (<https://jbi.global/covid-19#professionals>)

| **Search query** | **Date searched** | **# results retrieved** | **# results selected** |
| --- | --- | --- | --- |
| Faith OR Religion | 2023-10-16 | 0 | 0 |

**10 Can COVID Resources & Events** (<https://cancovid.ca/resources/?search=religion&source&topic> ; <https://cancovid.ca/events/>)

| **Search query** | **Date searched** | **# results retrieved** | **# results selected** |
| --- | --- | --- | --- |
| Faith OR Religion | 2023-10-16 | 0 | 0 |

### **11 OPHLA: Custom Search Engine for Ontario Public Health Unit Websites** (<https://www.ophla.ca/p/customsearchoph.html>)

| **Search query** | **Date searched** | **# results retrieved** | **# results selected** |
| --- | --- | --- | --- |
| Covid-19 AND vaccine, collaborat*, faith-based | 2022-03-07 | 64 | 0 |
| Covid-19 AND vaccine, collaborat*, faith-based | 2023-10-16 | 6 | X |

### **Library catalogs**

**1 PROQUEST- Public Health** (<https://www.proquest.com/>)

| **Search query** | **Date searched** | **#results retrieved** | **#results selected** |
| --- | --- | --- | --- |
| ab((vaccin* OR immuniz*) AND (faith* OR religio*) AND (implementation OR promotion)) | 2022-03-06 | 112 | 112 |
| Same search strategy as above | 2023-10-16 | 318 | 62 |

**2 OVID MEDLINE** (accessed through University of Toronto library)

| **Search query** | **Date searched** | **#results retrieved** | **#results selected** |
| --- | --- | --- | --- |
| description: "vaccine confidence" OR "vaccine uptake" OR "immunization" OR "vaccines" OR "vaccination" AND "faith communities" OR "faith-based organizations AND "engagement"  description: "vaccine confidence" OR "vaccine uptake" OR "immunization" OR "vaccines" OR "vaccination" AND "faith communities" OR "faith-based organizations AND "engagement" | 2022-02-07  2023-10-16 | 388  5 | 388  5 |

**3 ESBSCO-CINAHL** (accessed through University of Toronto library)

| **Search query** | **Date searched** | **# results retrieved** | **#results selected** |
| --- | --- | --- | --- |
| (MM: “vaccination: combined”) AND (MM: “collaboration”) OR (MM: “Faith-Based Organization”) OR (MM: church) OR (MM: “Evidence-Based”) OR (MM: “Systems Implementation”) | 2022-03-04 | 265 | 265 |
| (MM: “vaccination: combined”) AND (MM: “collaboration”) OR (MM: “Faith-Based Organization”) OR (MM: church) OR (MM: “Evidence-Based”) OR (MM: “Systems Implementation”) | 2023-10-16 | 2 | 0 |

**4 Cochrane Canada** (<https://www.cochranelibrary.com/search>)

| **Search query** | **Date searched** | **# results retrieved** | **#results selected** |
| --- | --- | --- | --- |
| (Vaccin* OR immuniz*) AND (faith* OR religio* OR church* OR temple* OR gurdwara* OR mosque) | 2022-02-07 | 1 | 1 |
| (Vaccin* OR immuniz*) AND (faith* OR religio* OR church* OR temple* OR gurdwara* OR mosque) | 2023-10-16 | 10 | 6 |

**5 SCOPUS- Conference paper** (<https://www.scopus.com/>)

| **Search query** | **Date searched** | **# results retrieved** | **#results selected** |
| --- | --- | --- | --- |
| ABS ( ( vaccin* OR immuniz* ) AND ( uptake OR intervention OR promotion OR implementation ) AND ( faith* OR religio* OR church* OR temple* OR gurdwara OR mosque ) ) AND ( LIMIT-TO ( PUBYEAR , 2022 ) OR LIMIT-TO ( PUBYEAR , 2021 ) OR LIMIT-TO ( PUBYEAR , 2020 ) OR LIMIT-TO ( PUBYEAR , 2019 ) OR LIMIT-TO ( PUBYEAR , 2018 ) OR LIMIT-TO ( PUBYEAR , 2017 ) OR LIMIT-TO ( PUBYEAR , 2016 ) OR LIMIT-TO ( PUBYEAR , 2015 ) OR LIMIT-TO ( PUBYEAR , 2014 ) OR LIMIT-TO ( PUBYEAR , 2013 ) OR LIMIT-TO ( PUBYEAR , 2012 ) OR LIMIT-TO ( PUBYEAR , 2011 ) ) AND ( EXCLUDE ( SUBJAREA , "BIOC" ) OR EXCLUDE ( SUBJAREA , "VETE" ) OR EXCLUDE ( SUBJAREA , "AGRI" ) OR EXCLUDE ( SUBJAREA , "ARTS" ) OR EXCLUDE ( SUBJAREA , "ENVI" ) OR EXCLUDE ( SUBJAREA , "COMP" ) OR EXCLUDE ( SUBJAREA , "MATH" ) OR EXCLUDE ( SUBJAREA , "CENG" ) OR EXCLUDE ( SUBJAREA , "DENT" ) OR EXCLUDE ( SUBJAREA , "ECON" ) OR EXCLUDE ( SUBJAREA , "ENGI" ) OR EXCLUDE ( SUBJAREA , "PHYS" ) ) | 2022-03-07 | 2 | 0 |
| CONF ( ( vaccin* OR immuniz* ) AND ( uptake OR intervention OR promotion OR implementation ) AND ( faith* OR religio* OR church* OR temple* OR gurdwara OR mosque ) ) AND ( EXCLUDE ( SUBJAREA , "BIOC" ) OR EXCLUDE ( SUBJAREA , "VETE" ) OR EXCLUDE ( SUBJAREA , "AGRI" ) OR EXCLUDE ( SUBJAREA , "ARTS" ) OR EXCLUDE ( SUBJAREA , "ENVI" ) OR EXCLUDE ( SUBJAREA , "COMP" ) OR EXCLUDE ( SUBJAREA , "MATH" ) OR EXCLUDE ( SUBJAREA , "CENG" ) OR EXCLUDE ( SUBJAREA , "DENT" ) OR EXCLUDE ( SUBJAREA , "ECON" ) OR EXCLUDE ( SUBJAREA , "ENGI" ) OR EXCLUDE ( SUBJAREA , "PHYS" ) ) AND ( LIMIT-TO ( PUBYEAR , 2023 ) OR LIMIT-TO ( PUBYEAR , 2022 ) ) | 2023-10-16 | 0 | 0 |

**6 SCOPUS- ALL** (<https://www.scopus.com/>)

| **Search query** | **Date searched** | **#results retrieved** | **#results selected** |
| --- | --- | --- | --- |
| ABS ( ( vaccin* OR immuniz* ) AND ( uptake OR intervention OR promotion OR implementation ) AND ( faith* OR religio* OR church* OR temple* OR gurdwara OR mosque ) ) AND ( LIMIT-TO ( PUBYEAR , 2022 ) OR LIMIT-TO ( PUBYEAR , 2021 ) OR LIMIT-TO ( PUBYEAR , 2020 ) OR LIMIT-TO ( PUBYEAR , 2019 ) OR LIMIT-TO ( PUBYEAR , 2018 ) OR LIMIT-TO ( PUBYEAR , 2017 ) OR LIMIT-TO ( PUBYEAR , 2016 ) OR LIMIT-TO ( PUBYEAR , 2015 ) OR LIMIT-TO ( PUBYEAR , 2014 ) OR LIMIT-TO ( PUBYEAR , 2013 ) OR LIMIT-TO ( PUBYEAR , 2012 ) OR LIMIT-TO ( PUBYEAR , 2011 ) ) AND ( EXCLUDE ( SUBJAREA , "BIOC" ) OR EXCLUDE ( SUBJAREA , "VETE" ) OR EXCLUDE ( SUBJAREA , "AGRI" ) OR EXCLUDE ( SUBJAREA , "ARTS" ) OR EXCLUDE ( SUBJAREA , "ENVI" ) OR EXCLUDE ( SUBJAREA , "COMP" ) OR EXCLUDE ( SUBJAREA , "MATH" ) OR EXCLUDE ( SUBJAREA , "CENG" ) OR EXCLUDE ( SUBJAREA , "DENT" ) OR EXCLUDE ( SUBJAREA , "ECON" ) OR EXCLUDE ( SUBJAREA , "ENGI" ) OR EXCLUDE ( SUBJAREA , "PHYS" ) ) | 2022-03-07 | 213 | 213 |
| ABS ( ( vaccin* OR immuniz* ) AND ( uptake OR intervention OR promotion OR implementation ) AND ( faith* OR religio* OR church* OR temple* OR gurdwara OR mosque ) ) AND ( LIMIT-TO ( PUBYEAR , 2022 ) OR LIMIT-TO ( PUBYEAR , 2023 ) OR EXCLUDE ( PUBYEAR , bioc OR exclude AND subjarea ) OR EXCLUDE ( PUBYEAR , agri OR exclude AND subjarea ) OR EXCLUDE ( PUBYEAR , envi OR exclude AND subjarea ) OR EXCLUDE ( PUBYEAR , math OR exclude AND subjarea ) OR EXCLUDE ( PUBYEAR , dent OR exclude AND subjarea ) OR EXCLUDE ( PUBYEAR , engi OR exclude AND subjarea ) ) | 2023-10-16 | 274 |  |

**7 PROQUEST - Policy File Index** (<https://about.proquest.com/en/products-services/policyfile/>)

| **Search query** | **Date searched** | **#results retrieved** | **#results selected** |
| --- | --- | --- | --- |
| (vaccin* OR immuniz*) AND (uptake OR intervention OR promotion OR implementation) AND (faith* OR religio* OR church* OR temple* OR gurdwara OR mosque) | 2022-03-07 | 0 | 0 |
| Vaccine AND (faith* OR religio*) | 2023-10-16 | 4 | X |
|  |  |  |  |

### **Web Searches**

**General** (<https://www.google.ca/>)

| **Search strategy** | **Date searched** | **# results retrieved** | **# results reviewed** | **# results selected** |
| --- | --- | --- | --- | --- |
| faith-based organization intervention, vaccine uptake and confidence, survey | 2022-02-07 | 6,810,000 | 100 | 22 |
| faith based collaboration vaccination | 2022-03-07 | 6,780,000 | 350 | 100 |
| faith-based collaboration, religious, vaccination | 2022-03-07 | 112,000 | 100 | 2 |
| Faith based AND vaccination AND community-based interventions | 2022-02-04 | 14,000 | 100 | 1 |
| faith based interventions OR faith based organizations, vaccine promotion | 2022-02-07 | 16,900 | 100 | 5 |
| faith based collaboration vaccination | 2023-10-16 | 58,500,000 | 100 | 39 |

### **Hand searched Journals**

| Journals | URL (if online) | Date searched | # results selected |
| --- | --- | --- | --- |
| **The Review of Faith and International Affairs** | <https://www.tandfonline.com/journals/rfia20> | 2022-02-07 | 1 |
|  | [All: faith] OR [[All: religio*] AND [All: vaccin*]] OR [[All: immuniz*] AND [All: intervention]] AND [in Journal: The Review of Faith & International Affairs] AND [Publication Date: (01/01/2022 TO 12/31/2023)] | 2023-10-16 | 1 |
| **Journal of Urban Health** | <https://journals.scholarsportal.info/browse/10993460>  Search term: "J Urban Health"[jour] (vaccin* OR immuniz*) AND (faith* OR religiou* OR spirit*) AND (intervention OR effectiveness) | 2022-03-08 | 0 |
|  | "J Urban Health"[jour] (vaccin* OR immuniz*) AND (faith* OR religiou* OR spirit*) AND (intervention OR effectiveness) | 2023-10-16 | 0 |
| **Journal of Religion and Health** | <https://link.springer.com/search?query=vaccine&search-within=Journal&facet-journal-id=10943> search term: vaccine* OR immunization | 2022-02-07 | 0 |
|  | <https://link.springer.com/search?query=vaccine&search-within=Journal&facet-journal-id=10943> search term: vaccine* OR immunization | 2023-10-16 | 0 |
| **Christian Journal for Global Health** | <https://journal.cjgh.org/index.php/cjgh/issue/archive> | 2022-03-22 | 1 |
|  | search term: vaccine* OR immunization | 2023-10-16 | 0 |
| **Journal of Immigrant and Minority Health** | <https://www.springer.com/journal/10903> (search term: description: "vaccine confidence" OR "vaccine uptake" OR "immunization" OR "vaccines" OR "vaccination" AND "faith-based organizations" AND "engagement") | 2022-03-22 | 1 |
|  | (search term: description: "vaccine confidence" OR "vaccine uptake" OR "immunization" OR "vaccines" OR "vaccination" AND "faith-based organizations" AND "engagement") | 2023-10-16 | 0 |
| **Social Science and Medicine** | <https://www.sciencedirect.com/journal/social-science-and-medicine> | 2022-01-28 | 1 |
|  | Search term description: vaccine confidence OR vaccine uptake OR immunization OR vaccination AND faith-based organizations OR faith 0R religious AND engagement | 2023-10-16 | 0 |

### **Reference lists scanned (n=52)**

| **Citation** | **#references scanned** | **#results selected** |
| --- | --- | --- |
| Dada, D., Djiometio, J. N., McFadden, S. M., Demeke, J., Vlahov, D., Wilton, L., ... & Nelson, L. E. (2022). Strategies That Promote Equity in COVID-19 Vaccine Uptake for Black Communities: a Review. *Journal of Urban Health*, 1-13. | 63 | 2 |
| Zampetakis, L. A., & Melas, C. (2021). The health belief model predicts vaccination intentions against COVID‐19: A survey experiment approach. *Applied Psychology: Health and Well‐Being*, *13*(2), 469–484. <https://doi.org/10.1111/aphw.12262> | all | 0 |
| Thomas, T. L., Strickland, O. L., DiClemente, R., Higgins, M., & Haber, M. (2012). Rural African American Parents’ Knowledge and Decisions About Human Papillomavirus Vaccination. *Journal of Nursing Scholarship*, *44*(4), 358–367. <https://doi.org/10.1111/j.1547-5069.2012.01479.x> | all | 0 |
| Mollers, M., Lubbers, K., Spoelstra, S. K., Weijmar-Schultz, W. C., Daemen, T., Westra, T. A., van der Sande, M. A., Nijman, H. W., de Melker, H. E., & Tami, A. (2014). Equity in human papilloma virus vaccination uptake?: sexual behaviour, knowledge and demographics in a cross-sectional study in (un)vaccinated girls in the Netherlands. *BMC Public Health*, *14*, 288–288. <https://doi.org/10.1186/1471-2458-14-288> | all | 0 |
| Lantos J. (2015). The patient-parent-pediatrician relationship: everyday ethics in the office. *Pediatrics in review*, *36*(1), 22–30. <https://doi.org/10.1542/pir.36-1-22> | all | 0 |
| van Lier, A., van de Kassteele, J., de Hoogh, P., Drijfhout, I., & de Melker, H. (2014). Vaccine uptake determinants in The Netherlands. *The European Journal of Public Health*, *24*(2), 304–309. <https://doi.org/10.1093/eurpub/ckt042> | all | 0 |
| Reñosa, M. D. C., Wachinger, J., Bärnighausen, K., Aligato, M. F., Landicho-Guevarra, J., Endoma, V., Landicho, J., Bravo, T. A., Demonteverde, M. P., Guevarra, J. R., de Claro III, N., Inobaya, M., Adam, M., Chase, R. P., & McMahon, S. A. (2021). How can human-centered design build a story-based video intervention that addresses vaccine hesitancy and bolsters vaccine confidence in the Philippines? A mixed method protocol for project SALUBONG. *BMJ Open*, *11*(6). <https://doi.org/10.1136/bmjopen-2020-046814> | all | 0 |
| Wondifon, E.A. (2013). Myths and Misconceptions as Barriers to Uptake of Immunization Services in Nigeria. *Journal of Vaccines and Vaccination, 04*. | all | 0 |
| Crocker-Buque, T., Edelstein, M., & Mounier-Jack, S. (2017). Interventions to reduce inequalities in vaccine uptake in children and adolescents aged <19 years: a systematic review. *Journal of Epidemiology and Community Health (1979-)*, *71*(1), 87–97. <http://resolver.scholarsportal.info.myaccess.library.utoronto.ca/resolve/0143005x/v71i0001/87_itriivaayasr.xml> | all | 0 |
| Derose, K. P., & Rodriguez, C. (2019). A Systematic Review of Church-Based Health Interventions Among Latinos. *Journal of Immigrant and Minority Health*, *22*(4), 795–815. <https://doi.org/10.1007/s10903-019-00941-2> | 1 | 0 |
| Tefera, Y. A., Wagner, A. L., Mekonen, E. B., Carlson, B. F., & Boulton, M. L. (2018). Predictors and Barriers to Full Vaccination among Children in Ethiopia. *Vaccines*, *6*(2). <https://doi.org/10.3390/vaccines6020022> | all | 0 |
| Korn, L., Böhm, R., Meier, N. W., & Betsch, C. (2020). Vaccination as a social contract. *Proceedings of the National Academy of Sciences of the United States of America*, *117*(26), 14890–14899. <https://doi.org/10.1073/pnas.1919666117> | all | 0 |
| Sharma, M., Batra, K., & Batra, R. (2021). A Theory-Based Analysis of COVID-19 Vaccine Hesitancy among African Americans in the United States: A Recent Evidence. *Healthcare*, *9*(10). <https://doi.org/10.3390/healthcare9101273> | all | 0 |
| Enea, V., Eisenbeck, N., Carreno, D. F., Douglas, K. M., Sutton, R. M., Agostini, M., Bélanger, J. J., Gützkow, B., Kreienkamp, J., Abakoumkin, G., Abdul Khaiyom, J. H., Ahmedi, V., Akkas, H., Almenara, C. A., Atta, M., Bagci, S. C., Basel, S., Berisha Kida, E., Bernardo, A., Buttrick, N. R., … Leander, N. P. (2022). Intentions to be Vaccinated Against COVID-19: The Role of Prosociality and Conspiracy Beliefs across 20 Countries. *Health communication*, 1–10. Advance online publication. <https://doi.org/10.1080/10410236.2021.2018179> | 3 | 0 |
| Dochez, C., Al Awaidy, S., Mohsni, E., Fahmy, K., & Bouskraoui, M. (2020). Strengthening national teams of experts to support HPV vaccine introduction in Eastern Mediterranean countries: Lessons learnt and recommendations from an international workshop. *Vaccine*, *38*(5), 1114–1119. <https://doi.org/10.1016/j.vaccine.2019.11.027> | 2 | 0 |
| Galang J. (2021). Science and religion for COVID-19 vaccine promotion. *Journal of public health (Oxford, England)*, *43*(3), e513–e514. <https://doi.org/10.1093/pubmed/fdab128> | 8 | 3 |
| Dhama, K., Sharun, K., Tiwari, R., Dhawan, M., Emran, T. B., Rabaan, A. A., & Alhumaid, S. (2021). COVID-19 vaccine hesitancy - reasons and solutions to achieve a successful global vaccination campaign to tackle the ongoing pandemic. Human vaccines & immunotherapeutics, 17(10), 3495–3499. <https://doi.org/10.1080/21645515.2021.1926183> | 5 | 1 |
| Corpuz J. (2021). Science, religion and state: a multidimensional perspective. *Journal of public health (Oxford, England)*, *43*(3), e547–e548. <https://doi.org/10.1093/pubmed/fdab152> | 5 | 1 |
| Jacobi, C. J., & Vaidyanathan, B. (2021). Racial differences in anticipated COVID-19 vaccine acceptance among religious populations in the US. *Vaccine*, *39*(43), 6351–6355. <https://doi.org/10.1016/j.vaccine.2021.09.005> | 12 | 0 |
| Babalola S. (2011). Maternal reasons for non-immunisation and partial immunisation in northern Nigeria. *Journal of paediatrics and child health*, *47*(5), 276–281. <https://doi.org/10.1111/j.1440-1754.2010.01956.x> | 1 | 0 |
| National Collaborating Centre for Methods and Tools. (2021, April 30). *Rapid Review: What is known about reasons for vaccine confidence and uptake in populations experiencing inequities?* <https://www.nccmt.ca/covid-19/covid-19-rapid-evidence-service/35> | 4 | 1 |
| Yong, A. G., Lemyre, L., Farrell, S. J., & Young, M. Y. (2016). Acculturation in Preventive Health for Immigrants: A Systematic Review on Influenza Vaccination Programs in a Socio-Ecological Framework. *Canadian Psychology / Psychologie Canadienne*, *57*(4), 340–355. <https://doi.org/10.1037/cap0000075> | 3 | 0 |
| Lee, M., Lim, H., Xavier, M. S., & Lee, E. Y. (2022). "A Divine Infection": A Systematic Review on the Roles of Religious Communities During the Early Stage of COVID-19. *Journal of religion and health*, *61*(1), 866–919. <https://doi.org/10.1007/s10943-021-01364-w> | 3 | 2 |
| Sevidzem Wirsiy, F. ., Nkfusai, N. C., Ebot Ako-Arrey, D., Kenfack Dongmo, E., Titu Manjong, F., & Nambile Cumber, S. (2021). Acceptability of COVID-19 Vaccine in Africa. *International Journal of Maternal and Child Health and AIDS (IJMA)*, *10*(1), 134–138. <https://doi.org/10.21106/ijma.482> | 3 | 1 |
| Wamai, R. G., Ayissi, C. A., Oduwo, G. O., Perlman, S., Welty, E., Manga, S., & Ogembo, J. G. (2012). Assessing the Effectiveness of a Community-Based Sensitization Strategy in Creating Awareness About HPV, Cervical Cancer and HPV Vaccine Among Parents in North West Cameroon. *Journal of Community Health*, *37*(5), 917–926. <https://doi.org/10.1007/s10900-012-9540-5> | 1 | 0 |
| Forster, A. S., Rockliffe, L., Chorley, A. J., Marlow, L. A., Bedford, H., Smith, S. G., & Waller, J. (2017). Ethnicity-specific factors influencing childhood immunisation decisions among Black and Asian Minority Ethnic groups in the UK: a systematic review of qualitative research. *Journal of Epidemiology and Community Health (1979-)*, *71*(6), 544–549. <https://doi.org/10.2307/44363660> | 3 | 0 |
| Khowaja, A. R., Khan, S. A., Nizam, N., Omer, S. B., & Zaidi, A. (2012). Parental perceptions surrounding polio and self-reported non-participation in polio supplementary immunization activities in Karachi, Pakistan: a mixed methods study. *Bulletin of the World Health Organization*, *90*(11), 822–830. <https://doi.org/10.2471/BLT.12.106260> | 3 | 0 |
| Levin J. (2014). Faith-based partnerships for population health: challenges, initiatives, and prospects. *Public health reports (Washington, D.C. : 1974)*, *129*(2), 127–131. <https://doi.org/10.1177/003335491412900205> | 4 | 1 |
| Olivier, J. (2014). *Scoping Review: Local Faith Communities And Immunization For Community And Health Systems Strengthening* (p. 103). The Joint Learning Initiative on Faith and Local Communities. [https://jliflc.com/wp-content/uploads/2014/09/Local-Faith-Communities-And-Immunization-For-Community-And-Health-Systems.pdf](https://jliflc.com/wp-content/uploads/2014/09/LOCAL-FAITH-COMMUNITIES-AND-IMMUNIZATION-FOR-COMMUNITY-AND-HEALTH-SYSTEMS.pdf) | 45 | 12 |
| Santibañez, S., Ottewell, A., Harper-Hardy, P., Ryan, E., Christensen, H., & Smith, N. (2022). A Rapid Survey of State and Territorial Public Health Partnerships With Faith-Based Organizations to Promote COVID-19 Vaccination. *American journal of public health*, *112*(3), 397–400. <https://doi.org/10.2105/AJPH.2021.306620> | 5 | 4 |
| Monguno A. K. (2013). Socio Cultural and Geographical Determinants of Child Immunisation in Borno State, Nigeria. *Journal of public health in Africa*, *4*(1), e10. <https://doi.org/10.4081/jphia.2013.e10> | 2 | 0 |
| Dubé, È., Ward, J. K., Verger, P., & MacDonald, N. E. (2021). Vaccine Hesitancy, Acceptance, and Anti-Vaccination: Trends and Future Prospects for Public Health. *Annual review of public health*, *42*, 175–191. <https://doi.org/10.1146/annurev-publhealth-090419-102240> | 4 | 0 |
| Levin, J., Idler, E. L., & VanderWeele, T. J. (2022). Faith-Based Organizations and SARS-CoV-2 Vaccination: Challenges and Recommendations. *Public health reports (Washington, D.C. : 1974)*, *137*(1), 11–16. <https://doi.org/10.1177/00333549211054079> | 50 | 6 |
| Idler, E., Levin, J., VanderWeele, T. J., & Khan, A. (2019). Partnerships Between Public Health Agencies and Faith Communities. *American journal of public health*, *109*(3), 346–347. <https://doi.org/10.2105/AJPH.2018.304941> | 7 | 3 |
| Williams, J., Miller, A., & Nussbaum, A. M. (2021). Combating Contagion and Injustice: The Shared Work for Public Health and Faith Communities During COVID-19. *Journal of religion and health*, *60*(3), 1436–1445. <https://doi.org/10.1007/s10943-021-01243-4> | 10 | 3 |
| Gunderson, G., & Cutts, T. (2021). Faith Communities as a Social Immune System: Recommendations for COVID-19 Response and Recovery. *Journal of Creative Communications*, *16*(2), 153–167. <https://doi.org/10.1177/0973258620983352> | 3 | 1 |
| Oman, D., & Lukoff, D. (2018). *Why Religion and Spirituality Matter for Public Health*. Springer International Publishing. <https://doi.org/10.1007/978-3-319-73966-3_13> | 7 | 0 |
| Nagar, S., & Ashaye, T. (2022). A Shot of Faith-Analyzing Vaccine Hesitancy in Certain Religious Communities in the United States. *American journal of health promotion : AJHP*, *36*(5), 765–767. <https://doi.org/10.1177/08901171211069547> | 8 | 4 |
| Ayton, D., Carey, G., Keleher, H., & Smith, B. (2012). Historical overview of church involvement in health and wellbeing in Australia: implications for health promotion partnerships. *Australian journal of primary health*, *18*(1), 4–10. <https://doi.org/10.1071/PY11079> | 1 | 0 |
| Dascalu S. (2019). Measles Epidemics in Romania: Lessons for Public Health and Future Policy. *Frontiers in public health*, *7*, 98. <https://doi.org/10.3389/fpubh.2019.00098> | 1 | 0 |
| Bogart, L., Dong, L., Gandhi, P., Ryan, S., Smith, T.L, Klein, D.J., Fuller, L and Ojikutu, B.O. (2021). What Contributes to COVID-19 Vaccine Hesitancy in Black Communities, and How Can It Be Addressed?. *RAND Corporation*. <https://www.rand.org/pubs/research_reports/RRA1110-1.html>  . | 1 | 0 |
| Bellatin, A., Hyder, A., Rao, S., Zhang, P. C., & McGahan, A. M. (2021). Overcoming vaccine deployment challenges among the hardest to reach: lessons from polio elimination in India. *BMJ global health*, *6*(4), e005125. <https://doi.org/10.1136/bmjgh-2021-005125> | 1 | 0 |
| Williams, J., Miller, A., & Nussbaum, A. M. (2021). Combating Contagion and Injustice: The Shared Work for Public Health and Faith Communities During COVID-19. *Journal of religion and health*, *60*(3), 1436–1445. <https://doi.org/10.1007/s10943-021-01243-4> | 7 | 3 |
| Ayub, S., Anugwom, G. O., Basiru, T., Sachdeva, V., Muhammad, N., Bachu, A., Trudeau, M., Gulati, G., Sullivan, A., Ahmed, S., & Jain, L. (2023). Bridging science and spirituality: The intersection of religion and public health in the COVID-19 pandemic. *Frontiers in Psychiatry, 14,* 1183234. <https://doi.org/10.3389/fpsyt.2023.1183234> | 55 | 0 |
| Mahachi, K., Kessels, J., Boateng, K., Jean Baptiste, A. E., Mitula, P., Ekeman, E., Nic Lochlainn, L., Rosewell, A., Sodha, S. V., Abela-Ridder, B., & Gabrielli, A. F. (2022). Zero- or missed-dose children in Nigeria: Contributing factors and interventions to overcome immunization service delivery challenges. *Vaccine, 40*(37), 5433–5444. https://doi.org/10.1016/j.vaccine.2022.07.058 | 137 | 0 |
| Melillo, S., Strachan, R., O’Brien, C. J., Wonodi, C., Bormet, M., & Fountain, D. (2022). Effects of Local Faith-Actor Engagement in the Uptake and Coverage of Immunization in Low- and Middle-Income Countries: A Literature Review. *Christian Journal for Global Health*, *9*(1), 2–32. <https://doi.org/10.15566/cjgh.v9i1.587> | all | 1 |
| Nicol, J. U., Iwu-Jaja, C. J., Hendricks, L., Nyasulu, P., & Young, T. (2022). The impact of faith-based organizations on maternal and child health care outcomes in Africa: Taking stock of research evidence. *The Pan African Medical Journal*, *43*(168), Article 168. <https://doi.org/10.11604/pamj.2022.43.168.32983> | all | 2 |
| Samsudin, K., Manaf, R. A., & Mahmud, A. (2023). Childhood Vaccine Hesitancy in selected Islamic and Muslim-majority Countries: Result Synthesis from a Scoping Review. *IIUM Medical Journal Malaysia*, *22*(3), Article 3. <https://doi.org/10.31436/imjm.v22i3.2179> | all | 0 |
| Syed, U., Kapera, O., Chandrasekhar, A., Baylor, B. T., Hassan, A., Magalhães, M., Meidany, F., Schenker, I., Messiah, S. E., & Bhatti, A. (2023). The Role of Faith-Based Organizations in Improving Vaccination Confidence & Addressing Vaccination Disparities to Help Improve Vaccine Uptake: A Systematic Review. *Vaccines*, *11*(2), 449. <https://doi.org/10.3390/vaccines11020449> | all |  |

**Fig 1**. PRISMA Flowchart

Records (academic and grey literature) identified through the literature search

(n=1361)

Titles/abstracts screened

(n=1127)

Full-text studies assessed for eligibility

(n=291)

Studies included in review

(n=160)

Identification

Screening

Eligibility

Inclusion

Duplicates removed

(n=234)

Titles/abstracts excluded

(n=836)

Full-text articles excluded:

(n=131)

• Did not have an intervention: n=39
• Did not involve more than two criteria: n=27

• Did not involve FBOs: n=19
• Did not involve public health agencies: n=18

• Did not involve vaccination: n=14

• Did not locate full text: n=8
• Outside inclusion data range: n=4

• Vaccine trial: n=2
