# Supplementary material for: Public health partnerships with faith-based organizations to support vaccination uptake among minoritized communities: A scoping review
Source: PLOS Glob Public Health. 2024 Jun 5;4(6):e0002765. doi: 10.1371/journal.pgph.0002765 (PMC11152308; doi:10.1371/journal.pgph.0002765)
Supplement: S3 File — (DOCX) [file pgph.0002765.s003.docx]

# Supporting Information 3. Document Extraction Codebook

| **Extraction Template Category** | **Definition, extraction instructions** |
| --- | --- |
| **General information** |  |
| Literature ID | Author, year. If no author, indicate publishing company/organization name and year published. |
| Type of Publication | [insert categories of publication here] |
| Title | Title of paper. |
| Lead Author | Full name of first author if present. |
| Country GDP | High-income, low-and-middle income, multiple high-income countries, multiple LMICs, or global. |
| Project Name | Insert project name. |
| **Collaborators** |  |
| FBO partners | Name all FBOs, including faith-based social service organizations, educational institutions, advocacy associations, charity, and places of worship mentioned in this intervention. |
| How are FBOs involved? | Indicate how and what roles did the FBOs play in supporting the vaccine uptake intervention. |
| Public Health partner involved | Name all Public Health Partners, including international non-governmental organizations, national and local public health agencies, departments of health. |
| How are PH involved? | Indicate how and what roles did the Public Health Partners play in supporting the vaccine uptake intervention. |
| Other agencies involved | Name all other agencies, including government institutions, research institutions, community-based organizations. |
| How are other agencies involved? | Indicate how and what roles did other agencies play in supporting the vaccine uptake intervention. |
| **Vaccine uptake interventions** |  |
| Vaccine type | From a multiple-answer checklist, select the vaccines that the intervention was designed to promote. |
| Intervention strategies | An 11-item multiple-answer checklist to select the types of intervention that took place as described in the article. Examples are: vaccine awareness, educational events, outreach, identification of target populations, generating guidelines and policies to support the intervention. |
| Detailed description of intervention strategies | This section is used to add explanations to the 11 items listed in the extraction item above. |
| Intervention mode | Describe the mode/platform through which the interventions were carried out (e.g., in-person faith-based locations, online, at home, at vaccination clinics, etc) |
| Religions involved | List the type of religions of faith-based organizations as described in the articles. One article may have more than one faith. |
| Study design | List the study design of the article. *In absence of a study design when the article is a news media report, the article will be extracted as a “case report (media)”. |
| Recruitment process | Describe how were the participants recruited to receive vaccine interventions. If the intervention was geared towards faith-leaders, describe how the faith leaders were recruited and by whom. |
| Intervention duration | The duration of the project, with start date and end date (if any). |
| Framework/model/theory used | List all the frameworks, models, and theories mentioned to support the design and implementation and evaluation of the vaccine intervention. |
| **Population and Participants** | |
| Baseline population characteristics | Briefly describe population demographic characteristics (gender, race, age, SES, religious majority) of the area of intervention if the article refers to it. (e.g., Bedouin women are the caretakers of children in a mainly nomadic society where most men are exposed to risk of contracting rabies from hunting wild boar in a mainly Sunni Islam tribe). |
| Intervention population | Select all key intervention populations from the multiple-answer checklist, i.e., rural, urban, immigrants and newcomers, populations in high-conflict areas, children, seniors, persons living with disability, people of faith, vaccine hesitant groups, etc. Items under this category may be modified after a 20-study extraction pilot. |
| Language of intervention(s) | All languages which the intervention was offered. |
| Number of participants recruited | Input number of participants who were contacted for the intervention (e.g., if 40 participants received consultation and 20 received a vaccination as a result of the consultation, input 40 here; if the intervention is towards 20 clergymen, input 20 clergyman here) |
| Number of participants who received the intervention | Input number of participants who received the intervention (e.g., if 40 participants received consultation and 20 received a vaccination because of the consultation, input 20 here) |
| Participant characteristics | Describe the gender, age, and characteristics of the recruited participants. |
| **Results** |  |
| Findings | List findings from the article related to vaccine uptake. |
| Limitations | List barriers and challenges of the vaccine intervention described in the article. |
| Comparison of population characteristics | Input characteristics of the participants in each row and interventions in each column in a matrix. Only applicable for interventions involving two or more intervention sites, populations, religions, etc. |
| Intervention outcome comparison | Input characteristics of the participants in each row and interventions in each column in a matrix. Only applicable for RCT's, CT's, interventions with temporal, multi-group, pre-post components. |
| Funding sources | List funding source and/or sponsor of the vaccine uptake intervention. |
| Future directions | Input any future research directions or intervention plans in the article, if any. |
