# Supplementary material for: Public health partnerships with faith-based organizations to support vaccination uptake among minoritized communities: A scoping review
Source: PLOS Glob Public Health. 2024 Jun 5;4(6):e0002765. doi: 10.1371/journal.pgph.0002765 (PMC11152308; doi:10.1371/journal.pgph.0002765)
Supplement: S4 File — (DOCX) [file pgph.0002765.s004.docx]

# Supporting Information 4. Religions and Languages reported in the vaccine initiatives

### Religions reported

| Religions | Count | (%) |
| --- | --- | --- |
| Christianity: | **160** | **48.3** |
| Christianity (denomination not specified) | 79 | 23.9 |
| Catholicism/Roman Catholicism | 33 | 10.0 |
| Evangelical Christianity | 6 | 1.8 |
| Mormonism | 3 | 0.9 |
| Baptist Church | 10 | 3.0 |
| United Church of the Christ | 2 | 0.6 |
| Anglicanism | 4 | 1.20 |
| Protestant (unspecified) | 6 | 1.8 |
| Adventist Protestant Christianism (Seventh day Adventism) | 1 | 0.3 |
| Orthodox Protestant | 1 | 0.3 |
| Anabaptism | 1 | 0.3 |
| Lutheranism | 3 | 0.9 |
| Ethiopian Orthodox Christianity | 1 | 0.3 |
| Methodism | 2 | 0.6 |
| African Methodist Episcopal | 1 | 0.3 |
| Jehovah's Witness | 1 | 0.3 |
| Orthodox Christianity | 3 | 0.9 |
| Presbyterian | 3 | 0.9 |
| Islam: | **74** | **22.4** |
| Islam (denomination not specified) | 73 | 22.1 |
| Sunni Islam | 1 | 0.3 |
| Judaism: | **23** | **6.9** |
| Judaism (denomination not specified) | 18 | 5.4 |
| Haredi Judaism | 1 | 0.3 |
| Orthodox Judaism | 4 | 1.2 |
| Hinduism | **15** | **4.5** |
| Buddhism: | **10** | **3.0** |
| Buddhism (denomination not specified) | 8 | 2.4 |
| Vajrayana Buddhism | 1 | 0.3 |
| Risshō Kōsei-kai | 1 | 0.3 |
| Sikhism | **3** | **0.9** |
| Baháʼí Faith | **1** | **0.3** |
| Not Specified | **45** | **13.6** |
| Total | **331** | **100.0** |

## Languages reported

| Languages | Count | (%) |
| --- | --- | --- |
| English | 113 | 39.2 |
| Spanish | 31 | 10.8 |
| Arabic | 8 | 2.8 |
| Urdu | 7 | 2.4 |
| Chinese | 4 | 1.4 |
| Korean | 4 | 1.4 |
| Somali | 4 | 1.4 |
| Filipino/Tagalog | 3 | 1.0 |
| Vietnamese | 3 | 1.0 |
| Yiddish | 3 | 1.0 |
| French | 3 | 1.0 |
| Amharic | 2 | 0.7 |
| Gujarati | 2 | 0.7 |
| Haitian | 2 | 0.7 |
| Hebrew | 2 | 0.7 |
| Karen | 2 | 0.7 |
| Kurdish | 2 | 0.7 |
| Malaysian | 2 | 0.7 |
| Mandarin | 2 | 0.7 |
| Marshallese | 2 | 0.7 |
| Pashto | 2 | 0.7 |
| Polish | 2 | 0.7 |
| Portuguese | 2 | 0.7 |
| Romanian | 2 | 0.7 |
| Russian | 2 | 0.7 |
| Tamil | 2 | 0.7 |
| Turkish | 2 | 0.7 |
| Bedouin Arabic | 1 | 0.3 |
| Bengali | 1 | 0.3 |
| Burmese | 1 | 0.3 |
| Dutch | 1 | 0.3 |
| Dzongkha | 1 | 0.3 |
| Fula | 1 | 0.3 |
| German | 1 | 0.3 |
| Hausa | 1 | 0.3 |
| Hindi | 1 | 0.3 |
| Hmong | 1 | 0.3 |
| Indigenous Language in Guatemala (not specified) | 1 | 0.3 |
| Krio (Sierra Leonean Creole) | 1 | 0.3 |
| Nepali | 1 | 0.3 |
| Nigerian Pidgin (Nigerian Creole) | 1 | 0.3 |
| Punjabi | 1 | 0.3 |
| Samoan | 1 | 0.3 |
| Susu | 1 | 0.3 |
| Swahili | 1 | 0.3 |
| Temne | 1 | 0.3 |
| Tongan | 1 | 0.3 |
| Welsh | 1 | 0.3 |
| Not Specified | 52 | 18.1 |
| Total | **288** | **100** |
