# Supplementary material for: Public health partnerships with faith-based organizations to support vaccination uptake among minoritized communities: A scoping review
Source: PLOS Glob Public Health. 2024 Jun 5;4(6):e0002765. doi: 10.1371/journal.pgph.0002765 (PMC11152308; doi:10.1371/journal.pgph.0002765)
Supplement: S5 File — (DOCX) [file pgph.0002765.s005.docx]

Supporting Information 5. Public Health Partners, Faith-Based Organization (FBO) Partners, and Other Partners

## Public Health Partners

| **Type of Organization** | **Examples** |
| --- | --- |
| **International Organizations** | |
| Intergovernmental Organizations (IGOs) | World Health Organization (WHO) [1–13]  WHO Information Network for Epidemics (WHO EPI-WIN) [14]  WHO-GPEI [15]  WHO/Europe [16]  UNICEF, Geneva Switzerland [3–7,12–15,17–23]  UNICEF - Global Polio Management Team (GPMT), Geneva, Switzerland [19]  Pan American Health Organization (PAHO), DC, USA; Guatemala City, Guatemala [6,8] |
| International Non-Governmental Organizations (INGOs) | GAVI, The Vaccine Alliance, Geneva, Switzerland [13,17,21,24,25]  Kenyan Health NGO Network (HENNET), Nairobi, Kenya [26]  Bill and Melinda Gates Foundation, WA, USA [2,10,21,27]  The International Vaccine Access Center, Maryland, USA [21]  International AIDS Vaccine Initiative, Multiple Locations in India, UK, US [21]  John Snow India Private Limited [11,28]  John Snow Inc., Arlington, USA [11,28] |
| **National** | |
| Ministries of health, public health agencies and their subsidiary departments/branches/institutes | National Health Services (NHS), England, UK [29–32]  Federal Ministry of Health, Nigeria [4,23]  Ministry of Health and Sanitation, Sierra Leone [33]  Ministry of Health of Liberia, Liberia [21]  Ministry of Public Health and Welfare, Guatemala (MSPAS) [6]  Ministry of Health-Ethiopia, Ethiopia [34]  Health Promotion Bureau (HPB), Ministry of Health, Sri Lanka [7]  The Zambia Ministry of Health [35]  Ministry of Health and Family Welfare (MoHFW) of India [11]  Ministry of Health of Zanzibar [36]  South Africa’s National Department of Health (NDoH) [37]  Public Health England, UK [18,29,31,38]  Public Health Scotland, UK [29]  Romanian National Institute of Public Health (INSP), Romania [39]  Centers for Disease Control and Prevention (CDC), Atlanta, GA, USA ([2,15,21,40–59]  CDC’s Racial and Ethnic Approaches to Community Health (REACH), USA [57]  Public Health Agency of Canada (PHAC), ON, Canada [60–62]  Department of Health and Social Care (DHSC), London, UK [29,63]  Immunisation and Countermeasures, National Infection Service, Public Health England, UK [31] Ghana Health Services, Accra, Ghana [64]  The U.S. Department of Health and Human Services (HHS), D.C, USA [21,41–44,54,65–74]  Health Resources and Services Administration (HRSA), HHS, MA, USA [54]  The Office of Minority Health of the U.S. Department of Health and Human Services, USA [75,76]  National Institute of Health (NIH), USA [40,77–79]  National Institute on Minority Health and Health Disparities, NIH, USA [80]  National Center for Advancing Translational Sciences of the National Institutes of Health (NIH), USA [75,76]  National Institutes of Health (NIH) Community Engagement Alliance Against COVID‐19 Disparities (CEAL), USA[65,81]  The National Primary Health Care Development Agency (NPHCDA), Nigeria [23,82]  The Department of Health in the Philippines, Manila, Philippines[83,84] |
| Other | White House Office Faith-Based and Neighborhood Partnerships, DC, USA [70–72] |
| **Provincial/State/Regional** | |
| State/provincial/regional departments of health, public health agencies, authorities, units | State Health Department of Kedah, Malaysia [85]  Washington State Department of Health, DDC, USA [86]  Southern Nationals & Nationalities Peoples (SNNP) Regional Health Bureau, Ethiopia [34]  Gambella Regional Health Bureau, Gambella, Ethiopia [34]  Arkansas Department of Health (ADH), AK, USA [87]  Kajiado County Department of Health Services and Public Health, Kajiado, Kenya [26]  Colorado Department of Public Health and Environment (CDPHE), CO, USA [53]  State of Tennessee Department of Health, TN, USA [88]  Virginia Department of Health (VDH), VA, USA [89]  New Jersey Department of Health, NJ, USA [90]  Pennsylvania Department of Health (Public Heath), PA, USA [91]  Massachusetts Department of Public Health, MA, USA [92,93]  DC Health, DC, USA [94]  Minnesota Department of Health, MN, USA [95] Victorian Department of Health, VIC, Australia [96]  Connecticut Department of Public Health, CT, USA [97]  Pennsylvania Department of Health (Public Heath), PA, USA [91] (  Salford Council Public Health, Salford, UK [29]  Municipal Health Services (Gemeentelijke Gezondheidsdienst, GGD), The Netherlands [98]  Western Sydney Public Health Unit, NSW, Australia [99]  State of Oregon Health Authority, OR, USA [73]  California Department of Public Health (CDPH), CA, USA [100]  Alberta Health Services (AHS), AB, Canada [101]  Pennsylvania Office of Rural Health (PORH), PA, USA [91]  Minnesota Public Health, MN, USA [18,49,102,103]  Lenoir County Health Department, NC, USA [104]  Los Angeles County Department of Health Services Office, CA, USA [105]  City of Detroit Dept. of Health, MI, USA [106]  Tri-County Health Department (TCHD), CO, USA [53]  Denver Public Health, CO, USA [53]  Region of Waterloo Public Health, ON, Canada [107]  Peel Public Health, ON, Canada [108]  Cincinnati Health Department, OH, USA [56]  Boston Public Health Commission, MA, USA [92]  Cleveland Department of Public Health, OH, USA [57]  The NYC Department of Health and Mental Hygiene, NY, USA [109]  NYC Health Departments, NY, USA [110]  Westchester County Department of Health, NY, USA [111] (=  County of Marin Department of Health and Human Services, CA, USA [112]  Berlin-Neukoelln local public health agency, Germany [113]  Toronto Public Health (TPH), ON, Canada [114]  The Southern Nevada Health District’s (SNHD) Office of Chronic Disease Prevention and Health Promotion, USA [57] |
| Government-sponsored State/Regional NGOs: including primary care provider networks and health providers focused on public health | Association of State and Territorial Health Officials (ASTHO), GA, USA [42,43,54,57,115]  Association of Immunization Managers (AIM), MD, USA [102]  Community Healthcare Network, NY, USA [116]  Centre for Infectious Disease Research in Zambia (CIDRZ), Lusaka, Zambia [85]  Development Research and Projects Center (dRPC), Federal Capital Territory, Nigeria [117]  Massachusetts Health Officers Association, MA, USA [93],  Bayelsa State Primary Healthcare Board, Bayelsa State, Nigeria [10]  Best Chance Network, SC, USA [58] |
| Immunization Coalitions and Networks | Immunize Canada, Ottawa, ON, Canada [62]  The Communication Initiative Network and Partnership, DC, USA [118]  Intermountain West HPV Vaccination Coalition (IWHVC), UT, USA [119]  Core Group Polio Project (CGPP)- Angola and India [4]  Immunize Nevada, NV, USA [120]  Nevada Minority Health and Equity Coalition (NMHEC), NV, USA [120]  Nevada Vaccine Equity Collaborative (NVEC), NV, USA [121] |

## Faith Based Partners

| **Type of Faith-Based Partners** | **Examples** |
| --- | --- |
| **International** | |
| Intergovernmental organization | The Organization of Islamic Cooperation (OIC), Jeddah, Saudi Arabia [22] |
| Faith-based INGOs for humanitarian aid | World Vision, Uxbridge, UK (Anglican) [2,4,8,21,25,122]  Anglican Alliance, UK (Anglican) [123]  International Catholic Migration Commission, Geneva, Switzerland (Catholic) [123]  Adventist Development Relief Agency, MD, USA (Adventist) [2]  Caritas Internationalis, Freiburg im Breisgau, Germany (Catholic) [21]  Catholic Relief Services, MD, USA (Catholic, a subsidiary of Caritas) [2,4,21]  The Salvation Army International, USA (Christian) [4,21,103]  Salvation Army World Services Office, DC, USA [4]  TearFund, Teddington, UK (Christian) [21]  Latter-day Saints Charities, UT, USA (Adventist) [6,25]  Muslim Aid, London, UK (Muslim) [3,14,25,29,33,44,62,65,74,82,107,108,117,124–127] |
| Interfaith INGOs | Religions for Peace, NY, USA [8,14,123,128]  Network for Religious and Traditional Peacemakers (NRTP), Helsinki, Finland [14,74]  Parliament of the World’s Religions, IL, USA [17]  World Faiths Development Dialogue (WFDD), DC, USA [21,25] |
| Ecumenical (inter-church) organizations | World Council of Churches (WCC), Grand-Saconnex, Switzerland [14,123,129]  Christian Conference of Asia (CCA), Chiang Mai, Thailand [129]  All Africa Conference of Churches (AACC), Nairobi, Kenya (Represents 204 churches in 43 countries) [129]  World Student Christian Federation (WSCF), Geneva, Switzerland (Orthodox, Protestant, Catholic, Pentecostal and Anglican) [129] |
| Advocacy Councils | World Jewish Congress, NY, USA (Judaism) [123] |
| Global Health Advocacies | IMA World Health, DC, USA (Lutheran) [33]  The Islamic Advisory Group (IAG) for Polio Eradication, Kabul, Pakistan [127] |
| Christian Communions | Grace Communion International, NC, USA (Evangelical) [78] |
| Ethics Council | The Pontifical Academy for Life, Rome, Italy (Catholic) [1] |
| Faith-based news agency and blogs | Religion Unplugged, TX, USA [130]  Redeeming Babel, USA [40,130] |
| Associations for clergymen | Conservative Jewish Movements Rabbinical Assembly, NY, USA [124] |
| Private Non-for-Profit Foundation | The Aga Khan Foundation, Portugal (Ismaili Islam) [21] |
| **National/Regional** | |
| National religious values advocacy | The National Latino Evangelical Coalition (NaLEC), FL, USA [41,78,105]  American Bible Society, PA, USA [78,105]  The Muslim Association of Canada (MAC), AB, Canada [108]  Islamic Society of North America (ISNA), IN, USA & ON, Canada [74,124]  Muhammadiyah, Yogyakarta, Indonesia [9] |
| National healthcare/health-oriented advocacy organizations | National Muslim Covid-19 Response Committee, Kenya [14]  Christian Health Association of Ghana (CHAG), Ghana [33]  Christian Health Association of Sierra Leone (CHASL), Sierra Leone [33]  British Islamic Medical Association (BIMA), UK [29,30]  Muslim Council of Britain (MCB) COVID Response Group, London, UK [29]  American Muslim Health Professionals (AMHP), USA [44,65]  The National Muslim Task Force, USA [65]  The National Black Muslim COVID Coalition, USA [65]  The National Catholic Cares Coalition (for vaccine equity), USA [50]  Nishkam Healthcare Trust, UK [32]  Churches Health Association of Zambia, Zambia [35,131] |
| National interfaith organizations | Faith In Action, CA, USA [105]  Mosaica Center for Inter-religious Cooperation, Israel [16]  Interfaith Youth Core (IFYC), IL, USA [44,66,72,132]  Inter-Religious Council of Sierra Leone (IRCSL), Sierra Leone [33]  Inter-Religious Council of Kenya, Kenya [128] |
| National religious advisory councils/regulatory bodies | The British Board of Scholars & Imams (BBSI), UK [29,38]  Mosques and Imams National Advisory Board (MINAB), UK [29,30,38]  Muslim Council of Britain (MCB), UK [29]  Muslim Council of Scotland, UK [29]  British Fatwa Council, UK [29]  Catholic Bishops' Conference of the Philippines, Philippines [83,84]  Zimbabwe Council of Churches, Zimbabwe [128]  Canadian Council of Imams, Canada [62]  The Sikh Gurudwara Prabandhak Committee, India [28] |
| National governmental bodies | National Fatwa Council, Malaysia [133]  The Ministry of Hajj and Awqaf (Ministry of Religious Affairs), Afghanistan [5]  Zhung Dratsang (central monastic body), Kingdom of Bhutan [127]  The State Islamic Religious Department, Malaysia [133]  Islamic Foundation Bangladesh, Bangladesh [27] |
| National social services, social justice, and humanitarian aid organizations | Cardinal Onaiyekan Foundation for Peace (COFP), Abuja, Nigeria [14]  Muslim Family Counseling Services (MFCS), Kumasi, Ghana [33]  Islamic Relief USA, VA, USA [21]  UK Islamic Mission (UKIM), London, UK [29]  Muslim Charities Forum (MCF), UK [29]  Karimia Institute, Nottingham, UK (Muslim) [29]  The Conference of National Black Churches (CNBC), GA, USA [50]  The Neighborhood Resilience Project [NRP], PA, USA [134]  Association of Islamic Charitable Projects of North America (AICP), PA, USA [125]  The National Black Church Initiative (NBCI), GA, USA [52]  Radha Swami Satsang, India [28]  Church’s Auxiliary for Social Action (CASA), India [11] |
| National Christian communions/associations | Church of Uganda, Uganda (Episcopal Anglican, member of WCC) [127]  The Church of Pentecost, Ghana (Pentecostal Evangelical) [127]  Ethiopian Evangelical Church Mekane Yesus, Ethiopia (Evangelical) [4,26]  Episcopal Church of Bangladesh, Bangladesh (Episcopal) [21]  Romanian Orthodox Church (ROC), Romania (Roman Orthodox) [39]  Evangelical clerical activism, USA [67]  Bangladesh Baptist Church Fellowship (BBCF), Bangladesh [27]  Christian Churches Together in the USA, MI, USA [105]  National Association of Evangelicals (NAE), USA [40,78,105,130]  The National African American Clergy Network, USA [105] |
| Regional religious advisory councils | The Southeastern Minnesota Synod of the Evangelical Lutheran Church of America (ELCA) (Somali and campus-focused), MN, USA [103]  Massachusetts Council of Churches, MA, USA [125]  Archdiocese of Los Angeles, CA, USA [50]  North Carolina's Council of Churches, NC, USA [74] |
| Local congregations^1^ | Washington National Cathedral, DC, USA (Episcopal) [71]  The King United Church of Christ, MO, USA [105]  The United Church of Christ, Cleveland, OH, USA (Protestant) [105]  Church of the Nazarene, Kansas, TX, USA [78]  ADAMS Muslim Community Centre, VA, USA (Muslim community focused) [124]  Cove City Church, OH, USA [57]  Hyo Shin Bible Presbyterian Church of NY, NY, USA [57]  St. Margaret’s Anglican Church, ON, Canada [114]  Asociación Comunidad Sefardí de Buenos Aires (ACISBA), Buenos Aires, Argentina [8] |
| Local ecumenical organizations | Project Bridges, DC, USA [105] |
| Regional hospitals/health services centres/health ministry/initiatives | Center of Excellence in Faith and Health, Methodist LeBonheur Healthcare, Methodist University Hospital, TN, USA [115]  Buddhist Tzu Chi Medical Foundation, CA, USA [115]  South Brooklyn Interfaith Coalition (Lutheran Health Care), NY, USA [115]  Penrose-St. Francis Health Mission, CO, USA [54]  Advocate Health Care - The Center for Faith and Community Health Transformation, IL, USA [42,43,49,54,115,135]  Hatzola Manchester (Jewish emergency medical service), Manchester, UK [31]  The Henry Ford Macomb Faith Community Nursing Network (FCCN), MN, USA [55,56]  Trusted FACE (Faith-based Activation for COVID Elimination), MN, USA [55,56]  First Ladies for Health, OH, USA [56]  Trinity Baptist Church (TBC) Health & Wellness Ministry, SC, USA [58] |
| Regional social service-oriented agencies/social justice oriented FBOs | The Connections Shelter Ministry (CSM), MN, USA [103]  Eternal Grace Ministries, AK, USA [87]  Catholic Social Services, AB, Canada [101,125]  IFSSA - Islamic Family and Social Services Association, AB, Canada [101]  Edmonton Mennonite Centre for Newcomers, AB, Canada [101]  The Boston Project Ministries, MA, USA [125]  YMCA of Greater Boston, MA, USA [48]  YWCA Malden, MA, USA [48]  YMCA Southeastern Massachusetts, MA, USA [48]  Leaving the Streets Ministries, Inc., MA, USA [103]  Youth Ministries Cameron House, CA, USA [100]  The Association of Islamic Charitable Projects Massachusetts (AICP), MA, USA [93]  Stair Step Foundation, MN, USA (Black community focused) [102]  Worcester Interfaith, MA, USA [125] |
| **Other** | |
| Faith leaders/traditional leaders^3^; community ambassador | Imams, Rabbis, Priests, Swamis, Pastors, Muslim Opinion leaders (MOL), Sheiks, Chiefs, religious scholars and theologians’ Faith Vaccine Engagement Teams, Toronto, ON, Canada [114] |
| Leadership development & consulting | The Skinner Institute, MA, USA [105]  FreedomRoad.us, USA [105] |
| Religious studies and research institutions in higher education; includes seminaries, religious educational institutions, and learning hubs | Joint Learning Initiative on Faith and Local Communities (JLIFLC), Georgetown’s Berkley Center for Religion, Peace, & World Affairs; DC, USA [122]  Office of Faith Community Health Promotion, Office for Community Engagement and Neighborhood Health Partnerships at the University of Illinois at Chicago, IL, USA [54]  Southern Baptist Theological Seminary, KY, USA [67]  Institutional Diversity and Community Engagement, Princeton Theological Seminary, NJ, USA [105]  Catholic Higher Education Institutions (HEI; major ministry of the Philippine Roman Catholic Church), Philippines [136]  Al-Salam Institute, London, UK [29]  Department of Public Health, Robbins College of Health and Human Sciences, Baylor University, TX, USA [64]  Department of Statistical Science, College of Arts & Sciences, Baylor University, TX, USA [64] |
| ^1^ Types of faith-based organizations are adapted from the Public Health and Faith Community Partnerships toolkit [137]  ^2^ Local congregations are numerous, some initiatives list up to 100 places of worship and some may not disclose which places were involved. These include churches, mosques, masjids, synagogues, and temples of various faiths.  ^3^ These are the 9 most frequently mentioned faith leaders and traditional leaders. | |

## Other Partners (Government, Community-Based Organizations (CBOs), non-Public Health NGOs, Research, and Industry)

| **Type of Other Partners** | **Examples** |
| --- | --- |
| **International** | |
| Inter-governmental Organization | Office of the United Nations High Commissioner for Refugees (OHCHR), the Refugee Agency, Geneva, Switzerland [6,129]  United Nations Foundation, Geneva, Switzerland [21]  United Nations Population Fund (UNFPA), NY, USA [6]  United Nations Agencies and Development Partners, Tanzania [12] |
| International Non-profit Organizations (INGOs) | Institute of Statelessness and Inclusion (ISI.org), the Netherlands [129]  Africare, DC, USA (African affairs) [4]  CARE International, UK (humanitarian agency against global poverty) [4]  Save the Children, UK (humanitarian aid for children) [4]  National Association of Municipalities (ANAM), Guatemala [6]  Plan International, Spain [4]  Canadian International Development Agency (CIDA) (now International Development Research Centre), Ottawa, ON, Canada [6]  Guatemalan Indigenous Development Fund/ Fondo de Desarrollo Indigena Guatemalteco (FODIGUA), Guatemala [6]  Foundation Alex/ Fundación de Guatemalteca para ninos con sordoceguera Alex (FUNDAL), Guatemala [6]  TSE, Supreme Electoral Tribunal, Guatemala [6]  RESOLVE, DC, USA [138]  Save the Children, London, UK [4,21]  Another Option (global, project extracted operated in Uganda) [33]  CARE (Cooperative Assistance and Relief Everywhere) - Ethiopia Branch [4]  Project Concern International, San Diego, CA, USA [4] (for CGPP India)  Family Health International (FHI 360), NC, USA [36,117]  BRAC, Bangladesh [27]  Project Last Mile, Dar Es Salaam, Tanzania [37] |
| International Financial Institutions | The World Bank – UN, DC, USA [21]  MCB Bank, UK [29]  FMO Development Bank, NL [29]  World Bank Pandemic Emergency Financing Facility [27] |
| International Non-Political organizations | Lions Clubs International [21]  Rotary International, IL, USA [2–4,15,21,22] |
| **National/Provincial/Regional Government** | |
| National government/government agencies | Government of Pakistan [2,19,139]  Government of Nigeria [2,19]  Government of DR Congo [2,19]  Government of India [2,19]  The State Islamic Religious Department of Malaysia (government) [133]  Guatemalan Social Security Institute (IGSS) [6]  Secretariat of Social Works of the First Lady (SESOP), Guatemala [6]  Romanian state officials, Romania [39]  The United States Census Bureau, USA [87]  The United States Secretary of Education, USA [89]  The Government and the King of Bhutan, Bhutan [140]  The Government of Tanzania (Minister of Foreign Affairs; Ministry of Finance and Planning and Ministry of State) [12]  U.S Departments of Agriculture, Commerce, Education, Housing and Urban Development, the Department of Labor, and the Department of Transportation, USA [70]  The Small Business Administration, USA [70]  AmeriCorps, USA [70]  Minnesota State Government, USA [95] |
| National Emergency Services/military efforts | Department of Homeland Security, Federal Emergency Management Agency (FEMA), DC, USA [73]  Magen David Adom (MDA), Israel's national emergency medical service, Israel [126] (an auxiliary of the Israel Defense Forces during war; affiliated with the Ben Gurion University)  The Israel Defense Forces' Home Front Command, Israel [126]  Departments of Homeland Security and Veterans Affairs [70]  U.S. Army Maryland National Guard's Vaccine Equity Task Force (VETF), MD, USA [138] |
| Government International Aid Agencies | Peace Corps, DC, USA [73]  The United States Agency for International Development (USAID), DC, USA [2,4,6,9,27,28,33,70,127,141],  USAID- Breakthrough ACTION Nigeria office [142]  USAID – MOMENTUM - Moving Integrated, Quality Maternal, Newborn, and Child Health and Family Planning and Reproductive Health Services to Scale [141]  Swedish Ministry for Foreign Affairs, Swedish International Development Agency (SIDA), Stockholm, Sweden [6] |
| Provincial/State government | The State of California Governor's Office, CA, USA [100]  Government of Uttar Pradesh, Uttar Pradesh, India [2]  Government of the District Columbia, USA [94] |
| Municipal or Regional Government/Councils | Health & Wellbeing of the Walsall Metropolitan Borough Council, UK [29]  Birmingham Council, UK [29]  City of Mankato, MN, USA [103]  City Lights, Minority Affair Council, Arkansas, USA [87]  City of Edmonton, Government of Alberta, Canada [101]  Virginia Department of Social Services VA, USA [89]  Minneapolis Public Housing Authority, MN, USA [95]  Office of Rural Health, SC, USA [58]  San Diego City Council, District 9, Community Empowerment, CA, USA [81] |
| Medical and healthcare regulatory authorities, associations | The Medicines and Healthcare Regulatory Board (MHRA), Department of Health and Social Care, Government of the UK, UK [38]  Legislative Assembly, UK [6]  The Association of Greater Manchester Authorities (AGMA) - local government association for Greater Manchester, UK [29]  Medical Committees, Leicester, UK [29]  Medical Society of the District of Columbia [94] |
| **National/Regional** | |
| Charitable Foundations | Kaiser Permanente East Bay Community Foundation, Oakland, CA, USA [50]  Robert Wood Johnson Foundation [42] |
| Healthcare professional advocacy organizations and platforms: Organizations of a professional nature, those that consist of healthcare workers, paramedics, and other professionals, and CHCs | Professional associations of physicians and nurses in Guatemala [6]  The American Academy of Pediatrics [21]  World Health Innovation Summit [29]  The Royal College of General Practitioners, UK [63]  Alliance for Healthier Communities, Ontario, Canada [60]  British Columbia Association of Community Health Centres (BCACHC), BC, Canada [60]  Canadian Association of Community Health Centres (CACHC), Ottawa, ON, Canada [60]  Manitoba Association of Community Health (MACHC), MB, Canada [60]  Nova Scotia Association of Community Health Centres (NSACHC), ON, Canada [60]  Association of Ontario Health Centres (AOHC), ON, Canada [60]  Black Doctors COVID Consortium, Philadelphia, USA [143]  The San Diego Black Nurses Association, CA, USA [144]  American Pharmacists Association, USA [59]  Collaboration on Social Science and Immunisation (COSSI), Australia [96] |
| Research institutions/centres and scientific societies/academic -practice partnerships | The Schlesinger Institute for Medical-Halachic Research (for Color Green Vaccination Initiative), Jerusalem, Israel (8)  African Medical and Research Foundation (AMREF), Ethiopia [4]  The Sabin Vaccine Institute, DC, USA [21,145]  Cornell University Cooperative Extension - New York City (CUCE-NYC), NY, USA [116]  Clinical and Translational Science Center, Weill Cornell Medicine, Cornell University, NY, USA [116]  Public Religion Research Institute (PRRI), DC, USA [132]  Development Research Project Centre, Kano, Democratic Republic of the Congo [117]  The Synthesis and Translation of Research and Innovations from Polio Eradication (STRIPE) consortium, MD, USA [5]  Swiss Tropical and Public Health institute (Swiss TPH), Switzerland [146]  Center for Family Health Research in Zambia (CFHRZ), Zambia [35]  The South Carolina Cancer Prevention and Control Research Network (SC-CPCRN), SC, USA [58]  Academic Public Health Corps, MA, USA [93] |
| Universities and Colleges | The Department of Family Medicine and UPMC McKeesport Family Medicine Residency program, the University of Pittsburgh, PA. USA [134]  Dalhousie University and IWK Health Centre, NS, Canada [62]  School of Nursing, Vanderbilt University, TN, USA [88]  Department. of primary and community care, Radboud University, Nijmegen, Netherlands [98]  Scientific Institute for Quality for Healthcare, Radboud University, Nijmegen, Netherlands [98]  Department of International Health, Johns Hopkins Bloomberg School of Public Health, International Vaccine Access Center, Baltimore, MD, USA [147]  New York Academy of Medicine (NYAM), NY, USA [148]  Program in Islamic Studies, Johns Hopkins University, MD, USA [44]  Syiah Kuala University, Banda Aceh, Indonesia [33]  Region IV Public Health Training Center (R4PHTC), Interfaith Health Program (IHP) Rollins School of Public Health, Emory University, GA, USA [54,77]  Office of Faith Community Health Promotion, University of Illinois, IL, USA [54]  International Islamic University; Islamic University Islamabad [22]  Historically black colleges and universities (HBCUs) in the US: Central Piedmont Community College, Davidson College, Johnson C. Smith University, Queens University of Charlotte, and Wingate University, USA [89]  Department of Medicine & School of Dentistry, Meharry Medical College [88]  The Office of Diversity, Inclusion, and Health Equity, Johns Hopkins School of Medicine, MD, USA [51]  Division of Pulmonary and Critical Care Medicine, Department of Medicine, Johns Hopkins School of Medicine, MD USA [51]  Office of Faith Community Health Promotion, Office for Community Engagement and Neighborhood Health, University of Illinois –IL, USA [132]  Centre for Health, Law & Society, University of Bristol Law School, Bristol, UK [31]  Department of Sociology & Anthropology, Hebrew University of Jerusalem, Israel [31]  Department of Global Health and Development, London School of Hygiene & Tropical Medicine, UK [31]  Division of Infection and Immunity, University College London, UK [31]  Clinical Research Department, Faculty of Infectious and Tropical Diseases, London School of Hygiene & Tropical Medicine, UK [31]  Hospital for Tropical Diseases, University College London Hospital, UK [31]  Centre for Mathematical Modelling of Infectious Diseases, London School of Hygiene & Tropical Medicine, London, UK [31]  The University of North Carolina at Chapel Hill, NC, USA [149]  University of Utah, Utah, USA [119]  Albert Einstein College of Medicine, NY, USA [56]  The University of Cincinnati College of Medicine, OH, USA [56]  School of Public Health, Kwame Nkrumah University of Science and Technology, Kumasi, Ghana [64]  International Vaccine Access Center, Johns Hopkins Bloomberg School of Public Health, Baltimore, MD, United States [13]  University of Nevada Las Vegas – The Nevada Institute for Children’s Research (NICRP), NV, USA [57]  Johns Hopkins Center for Communication Programs (CCP), MD, USA [142]  Arizona State University (ASU), AZ, USA [150]  Northern Arizona University (NAU), AZ, USA [150]  University of Arizona (UA), AZ, USA [150]  University of Arkansas for Medical Sciences (UAMS), AR, USA [75,76]  Projekt DEAL, Germany [113]  The University of Potsdam, Potsdam, Germany [113]  Universities in New York (unspecified), USA [111]  Niger Delta University, Amassoma, Nigeria [10]  UC San Diego, CA, USA [81]  Dow University of Health Sciences, Karachi, Pakistan [145]  Aga Khan University, Karachi, Pakistan [145]  University of New South Wales, Sydney, Australia [145]  The Australian National University, ACT, Canberra, Australia [145] |
| Non-profit organizations (NPOs) | ImpactIsrael, Israel [14]  The United Way (US and Canada) [46]  Community Organized Relief Effort (CORE- formerly the J/P Haitian Relief Organization, Hurricane relief advocacy), USA [77]  Pastoralist Concern Association of Ethiopia, Ethiopia [4]  Focus 1000, Sierra Leone ([26], child advocacy);  American Red Cross, USA [21]  RESULTS, USA (NPO to end poverty) [21]  The ONE Campaign, USA (NPO to end poverty and hunger in Africa) [21]  Good Grief Trust, UK [29]  Health Commons Solutions Lab, ON, Canada [151]  Ad Council, NY, USA [40]  Schuylkill County's VISION, Schuylkill, PA, US [54]  Edmonton's Food Bank, AB, Canada [101]  Parkside Business and Community in Partnership (PBCIP), NJ, USA [90]  211 Edmonton, AB, Canada [138]  Action for Healthy Communities Society of AB, Canada [101] [www.a4hc.ca](http://www.a4hc.ca)  Multicultural Health Brokers, AB, Canada [101] [www.mchb.org;](http://www.mchb.org)  American Lung Association, USA [89]  The City Retreat, Leicester, UK [29]  Grand Challenges Canada, ON, Canada [117]  Bal Umang Drishya Sanstha, New Delhi, India [13]  Enrichment Services Program (ESP), GA, USA [57]  Community Action Partnership of Kern (CAPK), Kern County, CA, USA [57]  Community Action Program for Central Arkansas—White County, AR, USA [57]  Palmetto Community Action Partnership – Berkeley County, SC, USA [57]  Latino Health Access, CA, USA [57]  ASIA's International Community Health Center (ICHC), Asian Services in Action (ASIA), OH, USA [57]  Partnership for a Healthy Lincoln (PHL), NE, USA [57]  Korean Community Services of Metropolitan New York, Inc. (KCS), NY, USA [57]  Health Resources in Action, MA, US [93]  Buffalo schools, USA [70]  AIDS Healthcare Foundation, CA, USA [58]  American Cancer Society, USA [58]  Karen Organization of San Diego, CA, USA [81]  Kupanda Kids, CA, USA [81]  Partnership for the Advancement of New Americans, CA, USA [81]  Refugee Health Unit/ UC San Diego Center for Community Health, CA, USA [81]  City Heights Latino Organizing Committee (COLCH), US San Diego Center for Community Health, CA, USA [81]  Somali Bantu Community of San Diego, CA, USA [81]  South Sudanese Community Center of San Diego, CA, USA [81]  The Humanity Movement, CA, USA [81]  Unity in the Community, CA, USA [81]  San Diego Youth Will, CA, USA [81]  The Marin Ministerial Alliance (Alliance), as part of the Southern Marin Community Response Team (Southern Marin CRT or SM CRT), CA, USA [112]  The Somali Parents Advocacy Center for Education (SPACE), MA, US [93]  Lumacare, ON, Canada [114] |
| Hospitals and medical centres (for-profit and non-profit) | Asian Health Services, Oakland, CA, USA [100]  Main Line Health, PA, USA [143]  The Friends of Harlem Hospital, NY, USA [46]  The Karen Hospital, Nairobi, Kenya [26]  Rush University Medical Centre, IL, USA [66]  Western Ontario Health Team and Thames Valley Family Health Team, ON, Canada [60]  The University of Pennsylvania Health System, PA, USA [143]  M Health Fairview (a healthcare brand, collaboration of UMinn Medical School, UMinn Physicians, and Fairview Health Services), MN, USA [54,95]  Henry Ford Health System, IL, USA [55,56,106]  Oxford University Hospitals NHS Foundation Trust, Oxfordshire, UK [29]  Lancashire Care Foundation Trust, NHS, UK [152]  Mercy Catholic Medical Center-Mercy Philadelphia, PA, USA [143]  Kaiser Permanente East Bay Community Foundation, Oakland, CA, USA [50]  University of North Carolina (UNC) Health Alliance, NC, USA [74]  Women's Health Specialist Centre, Francophone de Toronto [62]  BC Children's Hospital, BC, Canada [62]  The Ottawa Hospital, ON, Canada [62]  Hospital for Sick Children, ON, Canada [62]  Johns Hopkins Bayview Medical Center hospital, MD, USA [51]  University-based adolescent health clinic, University of North Carolina at Chapel Hill, NC, USA [149]  East London NHS Foundation Trust, London, UK [31]  Imperial College Healthcare NHS Trust, London, UK [31]  Cancer centers (unspecified), UT, USA (83)  Barts Health NHS Trust, London, UK [29]  Boston Medical Centre (BMC), MA, USA [92,153]  Mayo Clinic in Arizona, USA [150]  Hennepin Healthcare, MN, USA [95]  Wright Wellness Center, SC, USA [58]  PRISMA Health, SC, USA [58]  The Indus Hospital, Karachi, Pakistan [145] |
| Community Health Centres | The Bahn Health Centre at Bahn Refugee Camp in Nimba, Liberia [20]  Community Health Centres across Toronto: Centre francophone du Grand Toronto; Chatham Kent; Compass; Hamilton Urban Core; Regent Park; Seaway Valley; Somerset West; South Riverdale; TAIBU; Woolwich; Univi), Toronto, ON, Canada [61]  Open Door Health Clinics, WA, USA [103]  Lowell Community Health Center, MA, USA [54,115]  Community health clinics, UT, USA [119]  Canadian Native Friendship Centre, AB, Canada [101]  Black Creek Community Health Centre, ON, Canada [114]  TAIBU Community Health Centre, ON, Canada [114]  Lakeshore Area Multi-Service Project (LAMP), LAMP Community Health Centre, ON, Canada [114]  Scarborough Centre for Healthy Communities, ON, Canada [114] |
| Health and social services coalitions | Act Now Kenya - Medicin san Frontier, Kenya [26]  Grange Pavillion Project - partnership of Grangetown Community Action, Cardiff University, Cardiff Council, Wales, UK [29]  Ethiopian Civil Society Health Forum, Addis Ababa, Ethiopia [26]  The Healthy Community Partnership (HCP), MA, USA [51]  Medicine for the Greater Good (MGG), MA, USA [51] |
| COVID-specific collaboratives | COVID Collaborative - “It’s Up to You” campaign, USA [40]  Project Peach (Georgians Against COVID-19), GA, USA [77]  The Tennessee Community Engagement Alliance Against COVID-19, TN, USA [88]  Community Engagement Alliance (CEAL) Against COVID-19, led by the NIH, UK [65,77]  COVID-19 vaccination mobile strike teams, AK & TN, USA [87,88]  Association of American Medical Colleges (Vaccine Voices), DC, USA [55,56]  Communities RISE (Reach, Immunizations, System Change for Equity), USA [57] |
| National/Regional Social Services and Civil Society Organizations | Basic Integrated Rural Development Society (BIRDS), Pakistan [26]  Civil society organizations (unspecified), Namibia, Liberia [20]  Women’s network (unspecified), Namibia, Liberia [20]  Immigrant Settlement agencies (unspecified), Ontario, Canada [108]  Lenoir County Chapter National Association for the Advancement of Colored People (NAACP), NC, USA [104]  National Association for the Advancement of Colored People (NAACP), VA, USA [89]  Hugh O'Brian Youth (HOBY), AR, USA [87]  Edmonton Immigrant Services Association, AB, Canada [101]  John Humphrey Centre for Peace and Human Rights, AB, Canada [101]  Africa Centre, AB, Canada [101]  ASSIST Community Services Centre, AB, Canada [101]  Francophonie Albertaine Plurielle, AB, Canada [101]  Creating Hope Society, AB, Canada [101]  Coalition for Justice and Human Rights, AB, Canada [101]  Virginia Office on Volunteerism and Community Services, VA, USA [89]  African Diaspora for Justice, NJ, USA [90]  Hispanic/Latinx and immigrant community-based organizations (unspecified), TN, USA [88]  Social service agencies and community-based organizations (unspecified), USA [56,110,111,119]  Somali Community Resettlement Service, MN, US [95]  Council of Agencies Serving South Asians, ON, Canada [114] |
| **Private Sector** | |
| Private industries: including hospitals, family health teams, private sectors and news agencies, “social impact agencies”. | Quest Diagnostics, NY, USA [46]  Langer Research Associates, NYC, USA [40]  Private companies (unspecified), Guatemala [6]  Centre for the Action of Corporate Social Responsibility (CSR) of Guatemala, Guatemala [6]  Values Partnerships (Black-owned consulting firm), DC, USA [40,41,105,130]  Healthy Connections Inc., Arkansas, USA [87]  Asian and Hispanic Chambers of Commerce, VA, USA [89]  Unilever, London, UK [24]  Salesforce, CA, USA [24]  DHL, Bonn, Germany [24]  Public Square Strategies L.L.C., USA (public relations and consulting firm) [40]  Senior centres, USA (locations not specified) [48]  Toronto North Local Immigration Partnership Agency, ON, Canada [114]  Westchester County Department of Correction, NY, USA [111]  Corona Management Systems, Abuja, Nigeria [10]  Palmetto Dental Services, SC, USA [58] |
| News agencies | News agencies (unspecified): Romania [39], Israel [126], Guatemala [6]  Sunday Richmond Times-Dispatch, Virginia, USA [89]  Radio One network, USA [89]  Sahan Journal, MN, USA [95] |
| Pharmacies | Pharmacies: MN, USA [103]  Pharmacies: AB, Canada [138]  Walgreens, IL, USA [58] |

References

1. The Vatican. Note on the morality of using some anti-Covid-19 vaccines (21 December 2020) [Internet]. 2020 [cited 2022 Mar 25]. Available from: https://www.vatican.va/roman_curia/congregations/cfaith/documents/rc_con_cfaith_doc_20201221_nota-vaccini-anticovid_en.html.

2. Coates EA, Waisbord S, Awale J, Solomon R, Dey R. Successful polio eradication in Uttar Pradesh, India: the pivotal contribution of the Social Mobilization Network, an NGO/UNICEF collaboration. Glob Health Sci Pract. 2013;1(1):68–83.

3. Bellatin A, Hyder A, Rao S, Zhang PC, McGahan AM. Overcoming vaccine deployment challenges among the hardest to reach: lessons from polio elimination in India. BMJ Glob Health. 2021 Apr;6(4):e005125.

4. Murphy E. Social Mobilization: Lessons from the CORE Group Polio Project in Angola, Ethiopia, and India [Internet]. USAID, The CORE Group; 2012 Sep [cited 2022 Apr 11]. Available from: https://coregroup.org/wp-content/uploads/media-backup/Polio_Initiative/smreport-online.pdf

5. Owoaje E., Rahimi AO., Kalbarczyk A., Akinyemi O., Peters MA., Alonge OO. Conflict, community, and collaboration: shared implementation barriers and strategies in two polio endemic countries. BMC Public Health. 2020;20(Suppl 4):1178.

6. Flores A, Villeda JA, Rodríguez-Fernández R, Chévez AE, Barrera L, Tezaguic R, et al. Advocacy and resource mobilization for rubella elimination in Guatemala. J Infect Dis. 2011;204(suppl_2):S598–602.

7. Wijesinghe MSD, Ariyaratne VS, Gunawardana BMI, Rajapaksha RMNU, Weerasinghe WMPC, Gomez P, et al. Role of Religious Leaders in COVID-19 Prevention: A Community-Level Prevention Model in Sri Lanka. J Relig Health. 2022;61(1):687–702.

8. Pan American Health Organization. Webinar: Faith-based actors as promoters of vaccination - PAHO/WHO | Pan American Health Organization [Internet]. 2022 [cited 2023 Oct 17]. Available from: https://www.paho.org/en/events/webinar-faith-based-actors-promoters-vaccination

9. Boro E, Sapra T, Lavison JF de, Dalabona C, Ariyaratne V, Samsudin A. The Role and Impact of Faith-Based Organisations in the Management of and Response to COVID-19 in Low-Resource Settings: Policy & Practice Note. Relig Dev. 2022 Jun 16;1(1):132–45.

10. Kaduru CC, Mbagwu GC, Aadum DK, Eshikhena G, Idim GA, Ibe UF, et al. Using community theater to improve demand for vaccination services in the Niger Delta Region of Nigeria. BMC Proc. 2023 Jul 3;17(Suppl 7):6.

11. Soni GK, Seth S, Arora S, Singh K, Kumari A, Kanagat N, et al. Harnessing the Power of Collaboration to Expand the Coverage and Equity of COVID-19 Vaccinations in India: A Community Collaboration Model. Vaccines. 2023 Jun;11(6):1022.

12. WHO. Tanzania welcomes Ted Chaiban - United Republic of Tanzania | ReliefWeb [Internet]. 2022 [cited 2023 Oct 17]. Available from: https://reliefweb.int/report/united-republic-tanzania/tanzania-welcomes-ted-chaiban

13. Banerjee P, Seth R, Dhaliwal BK, Sullivan A, Qiayum Y, Thankachen B, et al. Vaccine acceptance in rural India: Engaging faith leaders as vaccine ambassadors. Front Public Health. 2022 Sep 20;10:979424.

14. Part 1- The role & impact of faith actors in overcoming barriers to COVID-19 vaccination. [Internet]. 2021 [cited 2022 Jul 15]. Available from: https://www.youtube.com/watch?v=IRtk6F8NLVA

15. Aylward B, Tangermann R. The global polio eradication initiative: lessons learned and prospects for success. Vaccine. 2011;29:D80–5.

16. WHO EU. Israel: Religious leaders bolster COVID-19 response with civil society and WHO/Europe support [Internet]. WHO. 2022 [cited 2022 Mar 26]. Available from: https://www.euro.who.int/en/countries/israel/news/news/2022/01/israel-religious-leaders-bolster-covid-19-response-with-civil-society-and-whoeurope-support

17. Quezada M, Chu A. Bringing the Faith Community Together for Global Vaccine Equity [Internet]. UNICEF USA. 2021 [cited 2022 Mar 26]. Available from: https://www.unicefusa.org/stories/bringing-faith-community-together-global-vaccine-equity/39098

18. National Academies of Sciences E, Division H and M, Health B on G, Threats F on M, Nicholson A, Minicucci C, et al. A Systems Approach to Increasing Vaccine Confidence and Uptake: Opportunities for Community-Based Strategies [Internet]. The Critical Public Health Value of Vaccines: Tackling Issues of Access and Hesitancy: Proceedings of a Workshop. National Academies Press (US); 2021 [cited 2022 Feb 7]. Available from: https://www.ncbi.nlm.nih.gov/books/NBK572621/

19. UNICEF. Polio Communications Quarterly Update - June 2011 [Internet]. The Communication Initiative Network. 2011 [cited 2022 Apr 11]. Available from: https://www.comminit.com/content/polio-communications-quarterly-update-june-2011

20. Bedford J, Chitnis K, Webber N, Dixon P, Limwame K, Elessawi R, et al. Community Engagement in Liberia: Routine Immunization Post-Ebola. J Health Commun. 2017 Aug 30;22(sup1):81–90.

21. Aylward L. Faith & Immunization: Past, Present and Potential Roles of Faith-Inspired Organizations [Internet]. GAVI Alliance; 2012 May [cited 2022 Mar 25] p. 21. Available from: https://berkleycenter.georgetown.edu/publications/faith-immunization-past-present-and-potential-roles-of-faith-inspired-organizations

22. Progress report August‐December 2012: Prime Minister’s Polio Monitoring & Coordination Cell. Islamabad, Prime MInister’s Secretariat [Internet]. 2012 [cited 2022 Apr 11]. Available from: https://www.endpolio.com.pk/images/reports/polio-final-report.pdf

23. Limaye RJ, Sara AB, Siddique AR, Vivas C, Malik S, Omonoju K. Interpersonal and community influences affecting childhood vaccination decision-making among Nigerian caregivers: Perceptions among frontline workers in Nigeria. J Child Health Care. 2019;23(3):403–14.

24. Global Solidarity Fund. The Vaccines for All High-Level Roundtable: Seizing the Moment for Collaboration - Global Solidarity Fund [Internet]. Global Solidarity Fund. 2021 [cited 2022 Mar 22]. Available from: https://www.globalsolidarityfund.org/the-vaccines-for-all-high-level-roundtable-seizing-the-moment-for-collaboration/

25. Marshall K. Faith in Vaccines [Internet]. GAVI The Vaccine Alliance. 2015 [cited 2022 Mar 22]. Available from: https://www.gavi.org/faith-in-vaccines

26. Catholic Relief Services. Civil Society Organization Platforms Contribute to National Immunization Programs - PROMISING PRACTICES 2012–2018 [Internet]. Catholic Relief Services; 2019 [cited 2022 Apr 6]. Available from: https://www.crs.org/sites/default/files/tools-research/promising_practices_a4_final_rev071119_online.pdf

27. Patwary MM. Faith leaders in the fight against the pandemic [Internet]. BMJ Global Health blog. 2023 [cited 2023 Oct 17]. Available from: https://blogs.bmj.com/bmjgh/2023/03/07/faith-leaders-in-the-fight-against-the-pandemic/

28. Soni GK, Bhatnagar A, Gupta A, Kumari A, Arora S, Seth S, et al. Engaging Faith-Based Organizations for Promoting the Uptake of COVID-19 Vaccine in India: A Case Study of a Multi-Faith Society. Vaccines. 2023 Apr 13;11(4):837.

29. British Islamic Medical Association. COVID-19 Vaccine Hub | British Islamic Medical Association [Internet]. 2021 [cited 2022 Apr 7]. Available from: https://britishima.org/operation-vaccination/hub/

30. Al Jazeera. UK imams, influencers counter COVID vaccine misinformation [Internet]. 2021 [cited 2022 Apr 6]. Available from: https://www.aljazeera.com/news/2021/1/22/uk-imams-mobilise-to-counter-covid-19-vaccine-disinformation

31. Kasstan B, Mounier-Jack S, Letley L, Gaskell KM, Roberts CH, Stone NRH, et al. Localising vaccination services: Qualitative insights on public health and minority group collaborations to co-deliver coronavirus vaccines. Vaccine. 2022 Mar 25;40(14):2226–32.

32. Singh H. How faith groups are supporting health and social care [Internet]. Religion Media Centre. 2023 [cited 2023 Oct 17]. Available from: https://religionmediacentre.org.uk/news/how-faith-groups-are-supporting-health-and-social-care/

33. Melillo S, Fountain D, Bormet M, O’Brien C. Promising Practices for Engaging Local Faith Actors to Promote COVID-19 Vaccination: Lessons Learned from Four Countries: Ghana, Indonesia, Sierra Leone, and Uganda [Internet]. USAID MOMENTUM Country and Global Leadership; 2021. Available from: https://usaidmomentum.org/wp-content/uploads/2021/12/GECO-519_MCGL-Policy-Brief-Country-Deep-Dives_Sec.508comp-1_0.pdf

34. Bayih G, Teklu A, Mekonnen ZA, Zelalem M, Tsedaw T, Tefera S, et al. The Implementation of Social and Behavior Change Communication Intervention to Improve Immunization Demand: A qualitative study in Awabel District, Northwest Ethiopia. Ethiop J Health Dev. 2021;35(3):49–55.

35. Micek K, Hester KA, Chanda C, Darwar R, Dounebaine B, Ellis AS, et al. Critical success factors for routine immunization performance: A case study of Zambia 2000 to 2018. Vaccine X. 2022 Aug 1;11:100166.

36. Lauro M. The Power of Religious Leaders to Drive COVID-19 Vaccine Demand Zanzibar [Internet]. Boost Community. 2022 [cited 2023 Oct 17]. Available from: https://brightspots.boostcommunity.org/the-power-of-religious-leaders-to-drive-covid-19-vaccine-demand-zanzibar

37. Project Last Mile. Digital and community influencers drive COVID-19 vaccine uptake in targeted South African populations. – PLM – Project Last Mile [Internet]. Project Last Mile. 2022 [cited 2023 Oct 17]. Available from: https://www.projectlastmile.com/digital-and-community-influencers-drive-covid-19-vaccine-uptake-in-targeted-south-african-populations/

38. The British Board of Scholars and Imams. Top Ten Questions Imams & Scholars get asked about vaccines [Internet]. BBSI; 2020 [cited 2022 May 2]. Available from: https://www.bdct.nhs.uk/wp-content/uploads/2021/01/BBSI-Vaccines-2020-11.1.21.pdf

39. Dascalu S, Flammer PG, Ghafari M, Henson SC, Nascimento R, Bonsall MB. Engaging Religious Institutions and Faith-Based Communities in Public Health Initiatives: A Case Study of the Romanian Orthodox Church During the COVID-19 Pandemic. Front Public Health [Internet]. 2021 [cited 2022 Mar 31];9. Available from: https://www.frontiersin.org/article/10.3389/fpubh.2021.768091

40. Larry Ross Communications. Ad Council & COVID Collaborative Enlist Network of Evangelical Leaders & Healthcare Professionals to reach Evangelicals with Trusted Information [Internet]. A. Larry Ross Communications. 2021 [cited 2022 Mar 22]. Available from: https://alarryross.com/christiansandvaccineblog/2021/4/13/ad-council-amp-covid-collaborative-enlist-network-of-evangelical-leaders-amp-healthcare-professionals-to-reach-evangelicals-with-trusted-information

41. Salguero GA, Hauer M. COVID-19 Vaccines and the Faith Community [Internet]. 2021 Apr 6 [cited 2022 Nov 18]. Available from: https://www.cfr.org/event/covid-19-vaccines-and-faith-community

42. Santibañez S, Ottewell A, Harper-Hardy P, Ryan E, Christensen H, Smith N. A Rapid Survey of State and Territorial Public Health Partnerships With Faith-Based Organizations to Promote COVID-19 Vaccination. Am J Public Health. 2022 Mar;112(3):397–400.

43. Santibañez S, Davis M, Avchen RN. CDC Engagement With Community and Faith-Based Organizations in Public Health Emergencies. Am J Public Health. 2019 Sep;109(Suppl 4):S274–6.

44. Johns Hopkins University. Faith in the Vaccine Project [Internet]. John Hopkins Krieger School of Arts & Science, Program in Islamic Studies. 2021 [cited 2022 Jun 8]. Available from: https://krieger.jhu.edu/islamic/community-connections/faith-in-the-vaccine/

45. User Guide for Faith-Based Leaders Toolkit [Internet]. 2021 [cited 2022 Mar 22]. Available from: https://wecandothis.hhs.gov/sites/default/files/2021-06/FBO%20Full%20Toolkit_English%2006.11.2021.pdf

46. Schonfeld J. Faith and the COVID-19 Vaccine: “Using the Black Church to Get the Word Out” [Internet]. Interfaith America. 2021 [cited 2022 Jun 8]. Available from: https://www.interfaithamerica.org/faith-and-the-covid-19-vaccine-using-the-black-church-to-get-the-word-out/

47. Bishop Noonan J. Immunization Policy – Diocese of Orlando, Florida [Internet]. 2014 [cited 2022 Mar 30]. Available from: https://www.orlandodiocese.org/ministries-offices/schools/schools-parent-information/schools-immunization-policy/

48. National Center for Immunization and Respiratory Diseases, Department of Homeland Security. Cybersecurity and Infrastructure Security Agency. COVID-19 vaccine communication toolkit for community-based organizations : getting started [Internet]. CDC. 2021 [cited 2022 Mar 29]. Available from: https://stacks.cdc.gov/view/cdc/100570

49. Peterson P, McNabb P, Maddali SR, Heath J, Santibañez S. Engaging Communities to Reach Immigrant and Minority Populations: The Minnesota Immunization Networking Initiative (MINI), 2006-2017. Public Health Rep. 2019 May;134(3):241–8.

50. Kaiser Permanente. Faith Leaders Use Trusted Voices to Encourage Vaccination [Internet]. 2021. Available from: https://about.kaiserpermanente.org/community-health/news/faith-leaders-use-trusted-voices-to-encourage-vaccination

51. Monson K., Oluyinka M., Negro D., Hughes N., Maydan D., Iqbal S., et al. Congregational COVID-19 Conversations: Utilization of Medical-Religious Partnerships During the SARS-CoV-2 Pandemic. J Relig Health. 2021;60(4):2353–61.

52. Evans A, Webster J., Flores G. Partnering With the Faith-Based Community to Address Disparities in COVID-19 Vaccination Rates and Outcomes Among US Black and Latino Populations. JAMA. 2021;326(7):609–10.

53. Marx GE, Burakoff A, Barnes M, Hite D, Metz A, Miller K, et al. Mumps outbreak in a Marshallese community—Denver metropolitan area, Colorado, 2016–2017. Morb Mortal Wkly Rep. 2018;67(41):1143.

54. Kiser M. Public Health Reaching out to the Faith Community: Why, How, and What Works | https://ihpemory.org [Internet]. 2016 Apr 4 [cited 2023 Jul 12]. Available from: https://ihpemory.org/public-health-reaching-out-to-the-faith-community-why-how-and-what-works/

55. Howley EK. Improving COVID-19 Vaccination Rates Through Partnerships with Faith-Based Organizations [Internet]. VaccineVoices. 2022 [cited 2023 Oct 17]. Available from: https://www.vaccinevoices.org/resources/article/improving-covid-19-vaccination-rates-through-partnerships-faith-based-organizations

56. Association of American Medical Colleges. Building Trust and Confidence Through Partnerships Grant Program [Internet]. Center For Health Justice. 2023 [cited 2023 Oct 17]. Available from: https://www.aamchealthjustice.org/our-work/trustworthiness/trust-grants

57. CDC. Partnering for Vaccine Equity: Stories from the Field| CDC [Internet]. 2023 [cited 2023 Oct 19]. Available from: https://www.cdc.gov/vaccines/health-equity/field-stories.html

58. Mathias W, Nichols KA, Golden-Wright J, Fairman CM, Felder TM, Workman L, et al. Implementation During a Pandemic: Findings, Successes, and Lessons Learned from Community Grantees. J Cancer Educ Off J Am Assoc Cancer Educ. 2023 Jun;38(3):957–62.

59. Meyer BA, Viskupič F, Wiltse DL. Pharmacists to partner with religious leaders to overcome vaccine hesitancy among Christians. J Am Pharm Assoc JAPhA. 2022;62(1):302–4.

60. Hsiung S, Bhandari M, Pham J. Equity, Community and Trust: Building vaccine confidence with marginalized populations [Internet]. 2021 [cited 2022 Mar 29]. Available from: https://www.childrenshealthcarecanada.ca/vaccination-conversations-poster-hall2/2022/1/19/equity-community-and-trust-building-vaccine-confidence-with-marginalized-populations

61. Alliance for Healthier Communities. Community Vaccination Promotion - Ontario (CVP-ON) | Alliance for Healthier Communities [Internet]. Alliance for Healthier Communities. 2022 [cited 2022 Mar 29]. Available from: https://www.allianceon.org/Community-Vaccination-Promotion-In-Ontario

62. Health Canada. Ask the experts COVID-19 vaccines questions: Safety, ingredients and side effects [Internet]. 2021 [cited 2022 Mar 22]. Available from: https://www.canada.ca/en/health-canada/services/video/ask-experts-covid-19-vaccines.html

63. Royal College of General Practitioners. GPs call for high-profile campaign backed by faith leaders and prominent figures from BAME communities to increase COVID-19 vaccine uptake [Internet]. 2021 [cited 2022 Apr 7]. Available from: https://www.rcgp.org.uk/about-us/news/2021/february/gps-call-for-high-profile-campaign-backed-by-faith-leaders.aspx

64. Asare M, Agyei-Baffour P, Koranteng A, Commeh ME, Fosu ES, Elizondo A, et al. Assessing the Efficacy of the 3R (Reframe, Reprioritize, and Reform) Communication Model to Increase HPV Vaccinations Acceptance in Ghana: Community-Based Intervention. Vaccines. 2023 May;11(5):890.

65. Syed S, Wajid A. Muslim Community Engagement Efforts to Tackle COVID-19 Vaccine Misinformation. Harvard Medical School Primary Care Review [Internet]. 2021 Apr 16 [cited 2022 Mar 22]; Available from: http://info.primarycare.hms.harvard.edu/review/muslim-community-engagement-efforts

66. Patel E, Giess ME, Raushenbush PB. Interfaith “vaccine ambassadors” take up Biden’s Month of Action. Religion News Service [Internet]. 2021 Jun 3 [cited 2022 Jun 8]; Available from: https://religionnews.com/2021/06/03/interfaith-vaccine-ambassadors-take-up-bidens-month-of-action/

67. Hoffman J. Clergy Preach Faith in the Covid Vaccine to Doubters. The New York Times [Internet]. 2021 Mar 14 [cited 2022 Mar 22]; Available from: https://www.nytimes.com/2021/03/14/health/clergy-covid-vaccine.html

68. U.S. Department of Health and Human Services. User Guide for Faith-Based Leaders Toolkit [Internet]. HHS COVID-19 Public Education Campaign. 2021 [cited 2022 Nov 17]. Available from: https://wecandothis.hhs.gov/resource/fbo-hispanic-latino-faith-based-leaders-toolkit

69. Cox H, Gebru Y, Horter L, Palomeque FS, Myers K, Stowell D, et al. New York State, New York City, New Jersey, Puerto Rico, and the US Virgin Islands’ Health Department Experiences Promoting Health Equity During the Initial COVID-19 Omicron Variant Period, 2021-2022. Health Secur. 2023 Sep;21(S1):S25–34.

70. The White House. FACT SHEET: Biden-Harris Administration Celebrates the Second Anniversary of the Reestablishment of the White House Office of Faith-Based and Neighborhood Partnerships [Internet]. The White House. 2023 [cited 2023 Oct 17]. Available from: https://www.whitehouse.gov/briefing-room/statements-releases/2023/02/17/fact-sheet-biden-harris-administration-celebrates-the-second-anniversary-of-the-reestablishment-of-the-white-house-office-of-faith-based-and-neighborhood-partnerships/

71. Lee AJ. 5 Ways Churches Can Play a Critical Role in Vaccination Efforts [Internet]. Lewis Center for Church Leadership. 2021 [cited 2022 Mar 22]. Available from: https://www.churchleadership.com/leading-ideas/5-ways-churches-can-play-a-critical-role-in-vaccination-efforts/

72. Jenkins J. Vaccine hesitancy declines among faith groups, spurred partly by religious appeals. The Salt Lake Tribune [Internet]. 2021 Aug 1; Available from: https://www.sltrib.com/religion/2021/07/28/vaccine-hesitancy/

73. Rogers M, Vick H, Dietch S, Washington KL, Downer GA. The Impact of Federal, Faith and Community Partnership on the Frontlines of COVID-19 Vaccine Distribution | FEMA.gov [Internet]. 2021 Jul 28. Available from: https://www.fema.gov/event/impact-federal-faith-and-community-partnership-frontlines-covid-19-vaccine-distribution

74. Shimron Y, Banks AM. Faith, medical leaders collaborate to get COVID-19 vaccine in arms of more people. Religion News Service [Internet]. 2021 Feb 18; Available from: https://religionnews.com/2021/02/18/faith-medical-leaders-collaborate-to-get-covid-19-vaccine-in-arms-of-more-people/

75. Purvis RS, Vincenzo JL, Spear M, Moore R, Patton SK, Callaghan-Koru J, et al. Factors Associated With Marshallese and Hispanic Adults’ Willingness to Receive a COVID-19 Booster Dose. J Prim Care Community Health. 2023 May 16;14:21501319231171440.

76. Vincenzo JL, Spear MJ, Moore R, Purvis RS, Patton SK, Callaghan-Koru J, et al. Reaching late adopters: factors influencing COVID-19 vaccination of Marshallese and Hispanic adults. BMC Public Health. 2023 Apr 3;23(1):631.

77. National Institutes of Health. An Inter-Faith Alliance to Stop COVID-19 [Internet]. Community Engagement Alliance. 2022 [cited 2022 Apr 29]. Available from: https://covid19community.nih.gov/An-Inter-Faith-Alliance-to-Stop-COVID-19

78. National Association of Evangelicals. Will Evangelical Leaders Receive the COVID-19 Vaccine? [Internet]. National Association of Evangelicals. 2021 [cited 2022 Mar 31]. Available from: https://www.nae.org/evangelical-leaders-covid-19-vaccine/

79. Lahijani AY, King AR, Gullatte MM, Hennink M, Bednarczyk RA. HPV Vaccine Promotion: The church as an agent of change. Soc Sci Med. 2021 Jan 1;268:113375.

80. Ma GX, Minsun M. Lee, Yin Tan, Hanlon AL, Ziding Feng, Shireman TI, et al. Efficacy of a community-based participatory and multilevel intervention to enhance hepatitis B virus screening and vaccination in underserved Korean Americans. Cancer 0008543X. 2018;124(5):973–82.

81. Stadnick NA, Cain KL, Oswald W, Watson P, Ibarra M, Lagoc R, et al. Co-creating a Theory of Change to advance COVID-19 testing and vaccine uptake in underserved communities. Health Serv Res. 2022 Jun;57 Suppl 1(Suppl 1):149–57.

82. Oku A, Oyo-Ita A, Glenton C, Fretheim A, Eteng G, Ames H, et al. Factors affecting the implementation of childhood vaccination communication strategies in Nigeria: a qualitative study. BMC Public Health. 2017;17(1):200.

83. CBCP: Ethical and Scientific Issues of COVID-19 Vaccinations by Rev. Fr. Nicanor Austriaco, OP [Internet]. 2021 [cited 2022 Mar 24]. Available from: https://www.youtube.com/watch?v=Xjmt0IlFI-0

84. Gopez JMW. Building public trust in COVID-19 vaccines through the Catholic Church in the Philippines. J Public Health. 2021 Feb 26;43(2):e330–1.

85. Wesevich A, Chipungu J, Mwale M, Bosomprah S, Chilengi R. Health promotion through existing community structures: A case of churches’ roles in promoting rotavirus vaccination in rural Zambia. J Prim Care Community Health. 2016;7(2):81–7.

86. Northwest Catholic. School immunization policy updated to reflect Catholic teaching [Internet]. Northwest Catholic. 2019 [cited 2022 Mar 30]. Available from: https://nwcatholic.org/news/northwest-catholic/school-immunization-policy-updated-to-reflect-catholic-teaching

87. Arkansas Department of Health. Faith-Based Outreach Arkansas Department of Health [Internet]. Arkansas Department of Health. 2021 [cited 2022 Mar 22]. Available from: https://www.healthy.arkansas.gov/programs-services/topics/faith-based-outreach

88. Alcendor DJ, Juarez PD, Matthews-Juarez P, Simon S, Nash C, Lewis K, et al. Meharry Medical College Mobile Vaccination Program: Implications for Increasing COVID-19 Vaccine Uptake among Minority Communities in Middle Tennessee. Vaccines. 2022;10(2):211.

89. Virginia Department of Health. Outreach to racial and ethnic populations encouraging vaccination [Internet]. CIDRAP. [cited 2022 Mar 22]. Available from: https://www.cidrap.umn.edu/practice/outreach-racial-and-ethnic-populations-encouraging-vaccination

90. Scott E. N.J. hosts ‘Grateful for the Shot’ vaccine clinics with faith-based organizations. WHYY [Internet]. 2021 May 22 [cited 2022 Mar 22]; Available from: https://whyy.org/articles/n-j-hosts-grateful-for-the-shot-vaccine-clinics-in-collaboration-with-faith-based-organizations/

91. Pennsylvania Office of Rural Health. Rural Pennsylvania COVID-19 Faith Toolkit [Internet]. [cited 2023 Nov 9]. Available from: https://www.porh.psu.edu/wp-content/uploads/Rural-Pennsylvania-COVID-19-Faith-Toolkit.pdf

92. Assoumou SA, Peterson A, Ginman E, James T, Pierre CM, Hamilton S, et al. Addressing Inequities in SARS-CoV-2 Vaccine Uptake: The Boston Medical Center Health System Experience. Ann Intern Med. 2022 Jun 21;175(6):879–84.

93. Yasmin S, Haque R, Kadambaya K, Maliha M, Sheikh M. Exploring How Public Health Partnerships with Community-Based Organizations (CBOs) can be Leveraged for Health Promotion and Community Health. Inq J Health Care Organ Provis Financ. 2022 Jan 1;59:00469580221139372.

94. Government of the District Columbia. District of Columbia COVID-19 Vaccination Plan [Internet]. 2021 May p. 58. Available from: https://coronavirus.dc.gov/sites/default/files/dc/sites/coronavirus/page_content/attachments/DC_COVID-19-Vaccination%20Plan_FINAL.pdf

95. Fazal A. Overcoming Covid Vaccine Hesitancy Among Minnesota’s Somali Muslims [Internet]. The Hastings Center. 2022 [cited 2023 Oct 17]. Available from: https://www.thehastingscenter.org/overcoming-covid-19-vaccine-hesitancy-among-minnesotas-somali-muslims/

96. Kaufman J, Overmars I, Leask J, Seale H, Chisholm M, Hart J, et al. Vaccine Champions Training Program: Empowering Community Leaders to Advocate for COVID-19 Vaccines. Vaccines. 2022 Nov 9;10(11):1893.

97. Connecticut Department of Public Health. Faith-based collaboration provides outreach in multiple languages [Internet]. CIDRAP - Center for Infectious Disease Research and Policy. [cited 2022 Mar 22]. Available from: https://www.cidrap.umn.edu/practice/faith-based-collaboration-provides-outreach-multiple-languages

98. Ruijs WL, Hautvast JL, Kerrar S, van der Velden K, Hulscher ME. The role of religious leaders in promoting acceptance of vaccination within a minority group: a qualitative study. BMC Public Health. 2013 May 28;13(1):511.

99. Scott N, Gabriel S, Sheppeard V, Peacock A, Scott C, Flego K, et al. Responding to a measles outbreak in a Pacific island community in western Sydney: community interviews led to church-based immunization clinics. West Pac Surveill Response J WPSAR. 2015;6(2):51.

100. A Vaccination Webinar Collaboration [Internet]. 2021 [cited 2022 Apr 5]. Available from: https://www.ekklesiae.org/blog/2021/7/1/a-vaccination-collaboration

101. Riebe N. Edmonton coalition helps faith leaders preach the truth about COVID-19 vaccines | CBC News. CBC [Internet]. 2021 Oct 20; Available from: https://www.cbc.ca/news/canada/edmonton/covid-19-ecrrc-1.6218905

102. Posy A, Mohamed S, Babington-Johnson A. Engaging Faith Leaders in Improving Vaccine Confidence Webinar [Internet]. Engaging Faith Leaders in Improving Vaccine Confidence Webinar; 2021 Apr 7 [cited 2022 Mar 29]; Online. Available from: https://www.immunizationmanagers.org/resources/vaccine-confidence-toolkit-webinar-series-webinar-4/

103. Minnesota Dept. of Health. Partnering with faith communities: Pillars of support during the COVID-19 response - COVID-19 Stories [Internet]. 2022 [cited 2022 Nov 17]. Available from: https://www.health.state.mn.us/diseases/coronavirus/stories/faith.html

104. Choi C. Faith-based organizations help with COVID-19 vaccine outreach. https://www.witn.com [Internet]. 2021 Feb 18 [cited 2022 Aug 5]; Available from: https://www.witn.com/2021/02/18/faith-based-organizations-help-with-covid-19-vaccine-outreach/

105. Ad Council. Hispanic Faith Community Toolkit - COVID-19 Vaccine Education Initiative [Internet]. Hispanic Faith Communities Toolkit. 2021. Available from: https://hispanicfaithvaccinetoolkit.org/

106. Hospital Partners with Faith-based Group to Vaccinate Muslim Community [Internet]. 2021 [cited 2022 Mar 22]. Available from: https://www.aha.org/advancing-health-podcast/2021-04-07-hospital-partners-faith-based-group-vaccinate-muslim-community

107. Region of Waterloo. Hear Stories From Our Community - YouTube [Internet]. YouTube. 2022 [cited 2022 Jul 2]. Available from: https://www.youtube.com/playlist?list=PLRVDwi_WScLmhZuCCh4-oyd07nf4-kUZo

108. Alqazzaz K. Opinion | Community organizations play a critical role in getting vaccines to the most vulnerable. The Hamilton Spectator [Internet]. 2021 May 20 [cited 2022 Mar 22]; Available from: https://www.thespec.com/opinion/contributors/2021/05/20/community-organizations-play-a-critical-role-in-getting-vaccines-to-the-most-vulnerable.html

109. Diallo F, Paulino L, Shiman LJ, Freeman K, Brooks B, Banson D, et al. Engaging businesses and faith-based organizations in public health interventions: Lessons learned from a COVID-19 and flu vaccine detailing program in the Northeast Bronx. Public Health Pract Oxf Engl. 2023 Jun;5:100353.

110. Ige O, Watkins J, Pham-Singer H, Dresser M, Maru D, Morse M. Embedding Health Equity in a Public Health Emergency Response: New York City’s Covid-19 Vaccination Experience. NEJM Catal [Internet]. 2023 Apr 28 [cited 2023 Oct 17];4(2). Available from: https://catalyst.nejm.org/doi/full/10.1056/CAT.22.0425

111. Kader F, Kruchten S, Campo M, Collica-Cox K, Davidson C, Wald A, et al. Dialogic Health Education to Reduce COVID-19 Disparities and Increase Health Literacy in Community and Correctional Settings: Protocol for a Two-Pronged Health Education Program. JMIR Res Protoc. 2022 Oct 21;11(10):e37713.

112. Thompkins F, Goldblum P, Lai T, Reynolds J, Lachter R, Mhatre PG, et al. Using Cross-Cultural Collaboration to Establish a Working Coalition for An Equitable COVID-19 Vaccine Program. J Humanist Psychol. 2023 Feb 16;00221678221140625.

113. Tjaden J, Haarmann E, Savaskan N. Experimental evidence on improving COVID-19 vaccine outreach among migrant communities on social media. Sci Rep. 2022 Sep 28;12(1):16256.

114. City of Toronto. COVID-19: Vaccine Engagement Teams Updates [Internet]. City of Toronto. City of Toronto; 2023 [cited 2023 Oct 16]. Available from: https://www.toronto.ca/community-people/health-wellness-care/health-programs-advice/respiratory-viruses/covid-19/covid-19-vaccines/covid-19-city-immunization-program/vaccine-engagement-teams-updates/

115. Emory University Interfaith Health Program. Faith-Based and Public Health Partnerships: Reaching Vulnerable Populations [Internet]. 2016 [cited 2022 Mar 22] p. 2. Available from: https://ihpemory.org/wp-content/uploads/2017/05/Emory-ASTHO-CDC-Report-2016.pdf

116. Parker C. Extension Connections Help Bring COVID-19 Vaccinations to NYC Neighborhoods [Internet]. Cornell CALS. 2021 [cited 2022 Mar 22]. Available from: https://cals.cornell.edu/news/extension-connections-help-bring-covid-19-vaccinations-nyc-neighborhoods

117. Walker JA, Hashim Y, Oranye N. Impact of Muslim opinion leaders’ training of healthcare providers on the uptake of MNCH services in Northern Nigeria. Glob Public Health. 2019;14(2):200–13.

118. The Communication Initiative Network. Polio Communications Quarterly Update: Trust [Internet]. The Communication Initiative Network. 2013 [cited 2022 Apr 11]. Available from: https://www.comminit.com/content/polio-communications-quarterly-update-trust

119. Warner EL., Fowler B., Martel L., Kepka D. Improving HPV Vaccination Through a Diverse Multi-state Coalition. J Community Health. 2017;42(5):911–20.

120. Immunize Nevada. Nevada Vaccine Equity Collaborative | Immunize Nevada [Internet]. 2021 [cited 2022 May 4]. Available from: https://www.immunizenevada.org/nevada-vaccine-equity-collaborative

121. Hoozer NV. Faith-Based Vaccine Outreach Underway to Reach Northern Nevada Latinos [Internet]. KUNR Public Radio. 2021 [cited 2022 Aug 5]. Available from: https://www.kunr.org/public-health/2021-07-30/faith-based-vaccine-outreach-underway-to-reach-northern-nevada-latinos

122. Banks AM. World Vision faces pandemic’s ‘perfect storm,’ seeks to meet US, global needs. Religion News Service [Internet]. 2021 Apr 9 [cited 2022 Apr 1]; Available from: https://religionnews.com/2021/04/09/world-vision-faces-pandemics-perfect-storm-seeks-to-meet-us-global-needs/

123. Kit RevDrS, Irvine D. Part 2 - The role & impact of faith actors in overcoming barriers to COVID-19 vaccination [Internet]. 2021 Jun 17 [cited 2022 Jun 22]. Available from: https://www.who.int/news-room/events/detail/2021/06/17/default-calendar/covid-19-vaccine-communications-webinar-series-co-convened-by-the-world-health-organization-unicef-and-religions-for-peace-webinar-2-part-2

124. Schonfeld J. Faith and the COVID-19 vaccine: “Muslims were among the first to believe in vaccines” [Internet]. Interfaith America. 2021 [cited 2022 Jun 8]. Available from: https://www.interfaithamerica.org/faith-and-the-covid-19-vaccine-muslims-were-among-the-first-to-believe-in-vaccines/

125. Commonwealth of Massachusetts. COVID-19 Vaccine Equity Initiative: Tailored community- and faith-based outreach and education | Mass.gov [Internet]. Mass.gov. 2021 [cited 2022 Mar 22]. Available from: https://www.mass.gov/info-details/covid-19-vaccine-equity-initiative-tailored-community-and-faith-based-outreach-and-education

126. Rosen B, Waitzberg R, Israeli A, Hartal M, Davidovitch N. Addressing vaccine hesitancy and access barriers to achieve persistent progress in Israel’s COVID-19 vaccination program. Isr J Health Policy Res. 2021 Aug 2;10(1):43.

127. Strategic Engagement of Religious Leaders in COVID Vaccination Webinar [Internet]. 2021 [cited 2022 Apr 29]. Available from: https://www.youtube.com/watch?v=wbi0ZAYRgMw

128. WHO. WHO, faith partners and national governments –supporting national responses to COVID-19 [Internet]. 2021. Available from: https://www.who.int/news/item/10-11-2021-who-faith-partners-and-national-governments-supporting-national-responses-to-covid-19

129. Webinar: “Covid-19 vaccination: how churches can ensure that stateless people are not left behind” [Internet]. 2021 [cited 2022 Jun 22]. Available from: https://www.youtube.com/watch?v=qR05pIKFCkQ

130. Bombino CL. These Evangelicals Are Equipping Church Leaders To Address COVID-19 Misinformation. Religion Unplugged [Internet]. 2022 Mar 21 [cited 2022 Mar 22]; Available from: https://religionunplugged.com/news/2022/3/21/these-evangelicals-are-equipping-church-leaders-to-address-covid-19-misinformation

131. Hester KA, Sakas Z, Ogutu EA, Dixit S, Ellis AS, Yang C, et al. Critical interventions for demand generation in Zambia, Nepal, and Senegal with regards to the 5C psychological antecedents of vaccination. Vaccine X. 2023 Aug 1;14:100341.

132. Jones RP, Patel E. Religion to the rescue: How appeals to faith can inspire people to get COVID vaccination. USA TODAY [Internet]. 2021 Aug 18 [cited 2022 Feb 7]; Available from: https://www.usatoday.com/story/opinion/2021/08/18/covid-and-religion-why-appeals-faith-can-increase-vaccinations/8120262002/

133. Chan HK, Soelar SA, Md Ali SM, Ahmad F, Abu Hassan MR. Trends in Vaccination Refusal in Children Under 2 Years of Age in Kedah, Malaysia: A 4-Year Review From 2013 to 2016. Asia Pac J Public Health. 2018;30(2):137–46.

134. Hedlin C. Pittsburgh’s Black churches dispel COVID myths, improve vaccine access. PublicSource [Internet]. 2021 Mar 18 [cited 2022 Mar 22]; Available from: http://www.publicsource.org/pittsburgh-black-churches-improve-access-to-covid-19-vaccine/

135. Kiser M, Lovelace K. A National Network of Public Health and Faith-Based Organizations to Increase Influenza Prevention Among Hard-to-Reach Populations. Am J Public Health. 2019 Mar;109(3):371–7.

136. Vicente NE, Cordero DA. In the service of the Filipino: the role of Catholic higher education institutions in promoting COVID-19 vaccines in the Philippines. J Public Health. 2021;43(2):e377–8.

137. Rollins School of Public Health. Public Health and Faith Community Partnerships: Model Practices to Increase Influenza Prevention Among Hard-to-Reach Populations [Internet]. 2014 [cited 2022 Jun 21]. Available from: https://ihpemory.org/ihp-programs/public-health-and-faith-community-partnerships/

138. Weaver B. Faith & Vaccines Launched to Support COVID-19 Vaccination Access and Uptake [Internet]. RESOLVE. 2021 [cited 2022 Mar 22]. Available from: https://www.resolve.ngo/blog/Faith--Vaccines-Launched-to-Support-COVID-19-Vaccination-Access-and-Uptake.htm

139. Khan TM, Chiau LM. Polio vaccination in Pakistan: by force or by volition? The Lancet. 2015 Oct 31;386(10005):1733.

140. Rocha ICN. Employing medical anthropology approach as an additional public health strategy in promoting COVID-19 vaccine acceptance in Bhutan. Int J Health Plann Manage. 2021;36(5):1943–6.

141. MOMENTUM. Promoting Vaccination: A Toolkit for Collaborating with Faith Communities [Internet]. USAID MOMENTUM. 2023 [cited 2023 Oct 17]. Available from: https://usaidmomentum.org/resource/promoting-vaccination-a-toolkit-for-collaborating-with-faith-communities/

142. Desmon S. Engaging Religious Leaders to Boost COVID-19 Vaccination - Johns Hopkins Center for Communication Programs [Internet]. 2022 [cited 2023 Oct 17]. Available from: https://ccp.jhu.edu/2022/05/02/religious-leaders-covid/, https://ccp.jhu.edu/2022/05/02/religious-leaders-covid/

143. Laughlin J. Faith leaders heed the call to help get coronavirus vaccines to more Black Philadelphians. https://www.inquirer.com [Internet]. 2021 Feb 11 [cited 2022 Mar 22]; Available from: https://www.inquirer.com/health/coronavirus/covid-vaccine-african-episcopal-church-philadelphia-20210211.html

144. Trageser C. How Churches Are Influencing Vaccine Decisions [Internet]. KPBS Public Media. 2021 [cited 2022 Mar 22]. Available from: https://www.kpbs.org/news/faith-spirituality/2021/04/20/churches-influencing-vaccine-decisions

145. Qasim R, Farooqui WA, Rahman A, Haroon R, Saleem M, Rafique M, et al. Community centred co-design methodology for designing and implementing socio-behavioural interventions to counter COVID-19 related misinformation among marginalized population living in the squatter settlements of Karachi, Pakistan: a methodology paper. BMC Proc. 2023 Jul 12;17(7):15.

146. Oyo-Ita A, Bosch-Capblanch X, Ross A, Oku A, Esu E, Ameh S, et al. Effects of engaging communities in decision-making and action through traditional and religious leaders on vaccination coverage in Cross River State, Nigeria: A cluster-randomised control trial. PLoS ONE [Internet]. 2021;16(4 April). Available from: https://www.scopus.com/inward/record.uri?eid=2-s2.0-85104244244&doi=10.1371%2fjournal.pone.0248236&partnerID=40&md5=3932aa7cc0defee4c527d34e5ded5846

147. Privor-Dumm L, King T. Community-based Strategies to Engage Pastors Can Help Address Vaccine Hesitancy and Health Disparities in Black Communities. J Health Commun. 2020 Oct 2;25(10):827–30.

148. Bond KT, Jones K, Ompad DC, Vlahov D. Resources and Interest Among Faith Based Organizations for Influenza Vaccination Programs. J Immigr Minor Health. 2013 Aug 1;15(4):758–63.

149. Cates JR, Ortiz R, Shafer A, Romocki LS, Coyne-Beasley T. Designing Messages to Motivate Parents To Get Their Preteenage Sons Vaccinated Against Human Papillomavirus. Perspect Sex Reprod Health. 2012 Feb 9;44(1):39–47.

150. Ignacio M, Oesterle S, Mercado M, Carver A, Lopez G, Wolfersteig W, et al. Narratives from African American/Black, American Indian/Alaska Native, and Hispanic/Latinx community members in Arizona to enhance COVID-19 vaccine and vaccination uptake. J Behav Med. 2023 Apr 1;46(1):140–52.

151. Health Commons Solutions Lab. Partnerships with faith-based communities to support vaccination An engagement toolkit [Internet]. 2022 [cited 2022 Mar 22]. Available from: https://docs.google.com/presentation/d/1C0e03YawdGIeNsEkyu4KyojWE41vB4d_nJUotpjEVGo

152. Landon C, Maru A. Hepatitis B: Raising awareness in the South Asian community in Preston. Br J Community Nurs. 2013;18(1):19–21.

153. Tjilos M, Tamlyn AL, Ragan EJ, Assoumou SA, Barnett KG, Martin P, et al. “Community members have more impact on their neighbors than celebrities”: leveraging community partnerships to build COVID-19 vaccine confidence. BMC Public Health. 2023 Feb 16;23(1):350.
