# Supplementary material for: Public health partnerships with faith-based organizations to support vaccination uptake among minoritized communities: A scoping review
Source: PLOS Glob Public Health. 2024 Jun 5;4(6):e0002765. doi: 10.1371/journal.pgph.0002765 (PMC11152308; doi:10.1371/journal.pgph.0002765)
Supplement: S6 File — (DOCX) [file pgph.0002765.s006.docx]

## Supporting Information 6. Lead Partners and Delivery Personnel

| **Lead Partner** | **Count** | **(%)** |
| --- | --- | --- |
| PH-led | 65^1^ | 37.4 |
| Co-led | 62^2^ | 35.6 |
| Research Institute | 21 | 12.1 |
| FBO-led | 15 | 8.6 |
| CBO-led | 6 | 3.4 |
| Unable to tell | 5 | 2.9 |
| Total | 174 | 100 |

| Intervention delivery personnel | Count | (%) |
| --- | --- | --- |
| Faith/religious leaders | 97 | 28.5 |
| Public health/global health professionals | 56 | 16.5 |
| Healthcare professionals | 48 | 14.1 |
| Research/training staff from academic/research institution | 31 | 9.1 |
| Community members^3^ | 36 | 10.6 |
| Staff and volunteers for NGOs and CBOs | 24 | 7.1 |
| Members of coalition/task force/network | 12 | 3.5 |
| Students | 8 | 2.4 |
| Government/political leaders and representatives | 11 | 3.2 |
| Not specified | 17 | 5.0 |
| Total^4^ | **340** | **100%** |
| ^1^ PH-led: Ten examples funded by the “White House Office of Faith-based and Neighborhood Partnerships”, led by the CDC, are counted as one program.  ^2^ Co-led: includes cases that have been co-led by more than one partnering organization. Six examples led by Emory University’s Rollin’s School of Public Health are counted as one program.  ^3^ Interventions where community outreach volunteers, vaccine promotion ambassadors played the most important role in increasing confidence and uptake.  ^4^ There are many more delivery personnel than initiatives because each initiative may have multiple arms of interventions. We counted the delivery personnel of each intervention for all initiatives included in the scoping review. | | |
